# Single—Not Double—3D-Aromaticity in an Oxidized *Closo* Icosahedral Dodecaiodo-Dodecaborate Cluster

**DOI:** 10.1021/jacs.3c07335

**Published:** 2023-09-20

**Authors:** Jordi Poater, Sílvia Escayola, Albert Poater, Francesc Teixidor, Henrik Ottosson, Clara Viñas, Miquel Solà

**Affiliations:** † Departament de Química Inorgànica i Orgànica & IQTCUB, Universitat de Barcelona, Martí i Franquès 1-11, 08028 Barcelona, Spain; ‡ ICREA, Pg. Lluís Companys 23, 08010 Barcelona, Spain; § Departament de Química, Institut de Química Computacional i Catàlisi, Universitat de Girona, C/Maria Aurèlia Capmany, 69, 17003 Girona, Catalonia Spain; ∥ Donostia International Physics Center (DIPC), 20018 Donostia, Euskadi Spain; ⊥ Institut de Ciència de Materials de Barcelona, Consejo Superior de Investigaciones Científicas, Campus Universitat Autònoma de Barcelona, 08193 Bellaterra, Spain; # Department of Chemistry - Ångström Laboratory, Uppsala University, 751 20 Uppsala, Sweden

## Abstract

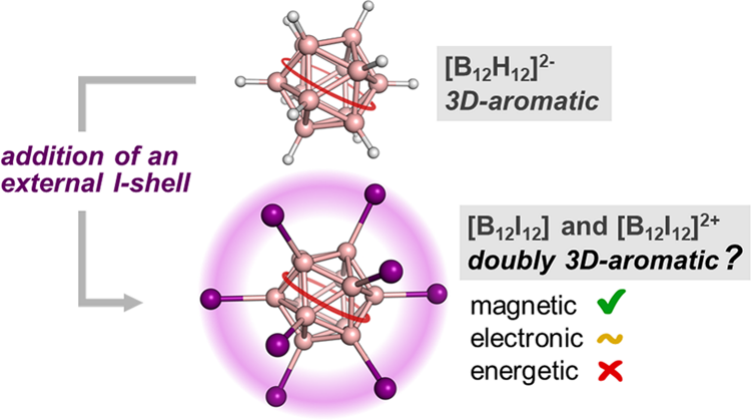

3D-aromatic molecules with (distorted) tetrahedral, octahedral, or spherical structures are much less common than typical 2D-aromatic species or even 2D-aromatic-in-3D systems. *Closo* boranes, [B_n_H_n_]^2–^ (5 ≤ *n* ≤ 14) and carboranes are examples of compounds that are singly 3D-aromatic, and we now explore if there are species that are doubly 3D-aromatic. The most widely known example of a species with double 2D-aromaticity is the hexaiodobenzene dication, [C_6_I_6_]^2+^. This species shows π-aromaticity in the benzene ring and σ-aromaticity in the outer ring formed by the iodine substituents. Inspired by the hexaiodobenzene dication example, in this work, we explore the potential for double 3D-aromaticity in [B_12_I_12_]^0/2+^. Our results based on magnetic and electronic descriptors of aromaticity together with ^11^B{^1^H} NMR experimental spectra of boron-iodinated *o*-carboranes suggest that these two oxidized forms of a *closo* icosahedral dodecaiodo-dodecaborate cluster, [B_12_I_12_] and [B_12_I_12_]^2+^, behave as doubly 3D-aromatic compounds. However, an evaluation of the energetic contribution of the potential double 3D-aromaticity through homodesmotic reactions shows that delocalization in the I_12_ shell, in contrast to the 10σ-electron I_6_^2+^ ring in the hexaiodobenzene dication, does not contribute to any stabilization of the system. Therefore, the [B_12_I_12_]^0/2+^ species cannot be considered as doubly 3D-aromatic.

## 1 Introduction

Doubly aromatic compounds are species that have two different (nearly) orthogonal types of aromaticity. The discovery of new doubly aromatic systems not only provides new insights into the fundamental principles of chemistry and chemical bonding but may also result in the development of new materials with enhanced stability and unique physicochemical properties. Double and even multiple (anti)aromaticity is frequent in the field of metallaaromatic compounds.^1−4^ For instance, LiAl_4_^–^ is doubly σ- and π-aromatic,^5^ Li_3_Al_4_^–^ in the singlet state is σ-aromatic and π-antiaromatic^6^ and in the triplet state is Hückel σ-aromatic and Baird π-aromatic,^7^ B_12_ has double σ- and π-disk aromaticity,^8^ B_6_^2–^ is doubly σ- and π-antiaromatic,^9,10^ Hf_3_ has triple σ-, π- and δ-aromaticity,^4,11^ V_2_B_7_^–^ is doubly σ/π aromatic,^12^ and Pa_2_B_2_ has been recently reported to be σ- and π-Möbius aromatic,^13^ among many other examples.

**Scheme 1:**
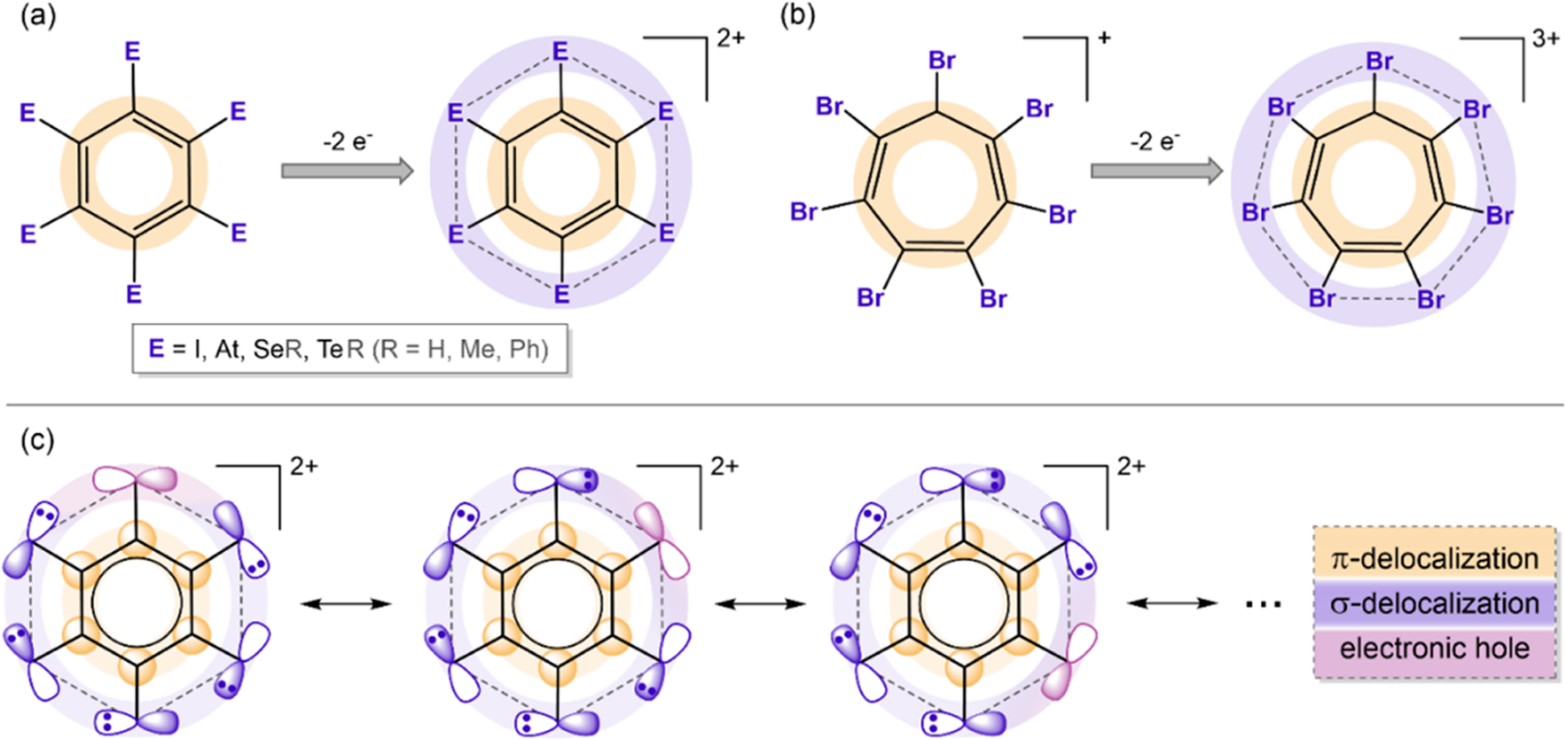
(a) Schematic Illustration of the Circular σ-Delocalization in Dicationic Hexahalo- or Hexachalco-Substituted Benzene Leading to Hückel σ-Aromaticity; (b) Schematic Representation of the Circular σ-Delocalization in Tricationic Hexabromotropylium Species in Its Lowest-Lying Triplet State Resulting in Baird σ-Aromaticity; (c) Double Aromaticity in C_6_E_6_^2+^ Requires the Opening of an Electronic Hole by Double Oxidation to Generate σ-Delocalization

Less common is the double aromaticity in organic compounds. Still, there are already a number of examples. The first reported doubly aromatic system was the 3,5-dehydrophenyl cation with σ- and π-aromaticity.^14^ However, the most well-known example of double aromaticity in organic chemistry is the singlet ground-state dication of hexaiodobenzene, [C_6_I_6_]^2+^ (see Scheme 1a), synthesized by Sagl and Martin.^15^ [C_6_I_6_]^2+^ is doubly Hückel σ- (with 10 delocalized electrons through the hexaiodo substituents) and π-aromatic (with six delocalized electrons in the benzene ring).^15,16^ Theoretical studies support the double σ- and π-aromaticity of [C_6_I_6_]^2+^^17^ as well as that of [C_6_At_6_]^2+^.^18^ For the former, relativistic effects are not so relevant but are essential for the latter.^19^ Moreover, several hexaselanylbenzene and hexatellanylbenzene dications [C_6_(ChR)_6_]^2+^ (Ch = Se, Te; R = H, Me, Ph) were found experimentally and computationally to be doubly aromatic compounds.^18−21^ Borazine analogues of hexaiodobenzene and hexakis(selenyl)benzene dication [B_3_N_3_I_6_]^2+^ as well as [B_3_N_3_(TeH)_6_]^2+^ were also reported to be doubly aromatic,^22^ although the π-aromaticity of borazine is known to be extensively lower than that of benzene.^23^ And very recently, the triplet state of [C_7_Br_7_]^3+^ has been found to be π-Hückel aromatic (with six π-electrons in the tropylium ring) and weakly σ-Baird aromatic (with 12 delocalized σ-electrons in the Br_7_ ring, Scheme 1b).^24^ Let us mention that Baird’s rule states that annulenes with 4*n* π-electrons are aromatic, and those with 4*n*+2 are antiaromatic in their lowest-lying triplet states.^25−27^ Other examples are the double σ- and π-aromaticity in bishomotriboriranide^28^ and in twisted thienylene–phenylene structures in toroidal and catenated topologies.^29,30^

It has to be mentioned that to reach double aromaticity in [C_6_I_6_]^2+^, double oxidation of C_6_I_6_ is required to open a hole in one of the 5p orbitals of iodine that generate six possible resonance structures (Scheme 1c depicts only three), if we consider only resonance structures in which the two electrons are removed from the same I atom, all of them with the same weight. The existence of these resonance structures generates the double aromaticity, and the fulfillment of Hückel’s rule in both the σ- and π-systems explains it.

Three-dimensional (3D) aromaticity has lately attracted increasing attention.^31−33^ The concept of 3D-aromaticity was introduced by King and Rouvray^34^ in 1977 and by Aihara^35^ in 1978 when they analyzed polyhedral boranes using a Hückel-type molecular orbital (MO) theoretical approach. 2D and 3D-aromaticity were perceived as two independent systems until 2014, when it was shown^36^ that the two were related, described as two sides of the same coin. In 2016, it was demonstrated^37^ that each aromatic annulene had its aromatic counterpart in the boron *closo* hydrides that could be categorized based on the Hückel’s 4*n* + 2 rule. Later on, some of us^38^ established the conditions that must be fulfilled for a compound to be 3D-aromatic, namely, (i) (at least) triply degenerate MOs as found in tetrahedral or higher symmetry molecules, (ii) a closed-shell electronic structure, which leads to a 6*n* + 2 electron count in the case of tetrahedral or octahedral molecules, (iii) extensive electron delocalization involving the complete 3D molecule leading to resonance stabilization, and (iv) similar (optoelectronic and magnetic) properties in the three *xyz* directions. Such conditions are obeyed only by a few compounds. The first known species with 3D-aromaticity were the c*loso* boranes, such as [B_10_H_10_]^2–^ or [B_12_H_12_]^2–^ and derivatives, synthesized at the end of the 1950s.^39,40^ Not only *closo* boranes [B_n_H_n_]^2–^ (5 ≤ *n* ≤ 14) but also their carboranes counterparts, which result from single or double substitution of BH by isoelectronic CH^+^ units in *closo* boranes, are 3D-aromatic compounds.^31^ These clusters obey the Wade’s 2*n* + 2 rule^41^ and the equivalent Mingos’ 4*n* + 2 rule.^42^ Their aromaticity is substantiated by results from nucleus-independent chemical shifts (NICS), bond length alternation (BLA) values, resonance energies, and ring currents.^34,35,37,43−46^ Moreover, the high thermal stability of these clusters^47−49^ further supports their aromatic character.^47,50,51^ A number of unique materials and applications were developed based on 3D-aromatic boranes, carboranes, and metallacarboranes due to their enhanced stability.^52−67^

With the present investigation, we would like to provide both evidence and counter-evidence of the potential double 3D-aromaticity of the dodecaiodo-dodecaborate cluster. There are several reasons, more exactly seven, for choosing [B_12_I_12_]^2–^. First, [B_12_H_12_]^2–^, which has the same boron cage as [B_12_I_12_]^2–^, is likely the most aromatic c*loso* borane.^37,68^ Indeed, it is the one that has the highest heat of formation among [B*_n_*H*_n_*]^2–^ (5 ≤ *n* ≤ 12) clusters,^69^ it has large adiabatic and vertical electron detachment energies, which is an indication of its high stability,^70^ it has the lowest average energy on a per-vertex basis,^44^ and it has the highest resonance energy.^35^ Second, [B_12_I_12_]^2–^ (and, in general all *closo* [B_12_X_12_]^2–^; X = halogen) has a high structural and electronic stability and chemical inertness, especially in front of electrophilic attacks, as well as superweak basicity.^71,72^ Third, [B_12_I_12_]^2–^ has the largest oxidation potential among all *closo* [B_12_X_12_]^2–^ (X = halogen).^73^ Fourth, the second electron in the double oxidation of *closo* [B_12_X_12_]^2–^ (X = F, Cl) is taken from the boron cage, whereas for X = I is taken from the halogen shell (for X = Br, we have an intermediate situation with the electron partially removed from the boron cage and the bromide shell).^72^ Remember that we need to open a hole in one of the *np* orbitals of perhalogenated *closo* [B_12_X_12_]^2–^, and this is possible only in *closo* [B_12_I_12_]^2–^. Fifth, because of the X···X distances, we expect to have an appreciable overlap between the *n*p orbitals only for [B_12_I_12_]^2–^. Such overlap is needed to allow electron delocalization in the I_12_ shell. Sixth, the I···I antibonding HOMO of [B_12_I_12_]^2–^ is triply degenerated (see Figure S1) as required for 3D-aromaticity. And finally, the seventh reason, the total number of electrons participating in lone pairs in the I_12_ shell is 72 electrons, a number that follows the 2(*n*+1)^2^ Hirsch rule^74^ for spherical aromaticity, with *n* = 5. However, the required oxidation to open a hole changes this number of electrons.

## 2 Methodology

All DFT calculations were performed with the Amsterdam Density Functional (ADF) program^75,76^ using dispersion-corrected density functional theory (DFT) with relativistic corrections at the ZORA-BLYP-D3(BJ)/TZ2P level of theory^77−80^ for geometry optimizations and energy calculations, with the full electron model for all atoms (no frozen core). All stationary points were verified to be minima on the potential energy surface through vibrational analysis.

The quantification of aromaticity is usually based on the fact that aromatic molecules have most of these properties: (i) an uninterrupted electron delocalization in a 2D or 3D-closed circuit,^81,82^ (ii) more stability than a nonaromatic reference compound,^83^ (iii) bond length equalization,^84^ and (iv) special response in the presence of an external magnetic field.^85−87^ It has to be stressed that energetic stabilization and electron delocalization are the two most important requirements for aromaticity, and the two are compulsory conditions, whereas ring currents and bond length equalization are usual conditions followed by most of the aromatic species, but not sufficient to prove aromaticity. We considered magnetic, electronic, and energetic descriptors of aromaticity since it is generally accepted that one should use a set of indices based on different properties to discuss the aromaticity of a given species.^88^

The magnetic properties of aromaticity were first evaluated by means of the nucleus-independent chemical shift (NICS), proposed by Schleyer and co-workers, as a magnetic descriptor of aromaticity.^89^ NICS is defined as the negative value of the absolute shielding computed at a ring center or at some other point in the system. Rings with large negative NICS values are considered aromatic. NICS values were computed using the gauge-including atomic orbital method (GIAO).^86,90^ NICS scans were carried out with the same ZORA-BLYP-D3(BJ)/TZ2P method. To analyze the importance of relativistic effects, we also computed the NICS scans of [B_12_I_12_]^2–^ and singlet [B_12_I_12_] clusters as well as those of C_6_I_6_ and C_6_I_6_^2+^ with the B3LYP/6-311++G**∼LANL2DZ method (Figures S21 and S22). Results show that, except for C_6_I_6_^2+^, there is an increase in the absolute NICS values (by less than 5 ppm) at the B3LYP/6-311++G**∼LANL2DZ level of theory, but when we compared the initial and the oxidized species, the trends remain the same. On the other hand, magnetically induced current-density susceptibility was obtained with gauge-including magnetically induced currents (GIMIC) method.^87,91^ GIMIC requires the basis set information, the atomic orbital density matrix, and the perturbed atomic orbital density matrices as input data, which have been obtained by performing NMR shielding calculations with the Gaussian 16 program package.^92^ The specific parameters used in the GIMIC calculations are summarized in Table S1. The NMR shielding calculations were performed with the B3LYP functional and the 6-311++G** basis set for boron and LANL2DZ basis set and pseudopotential for iodine using the ZORA-BLYP-D3(BJ)/TZ2P optimized geometries. Finally, the visualization of the current densities was carried out using ParaView 5.9.0.^93^

Second, aromaticity was also assessed through electron delocalization properties with the electron density of delocalized bonds (EDDB_G_) function.^94−96^ The required information on the formatted checkpoint file and the density matrix within the representation of natural atomic orbitals (NAOs) was obtained using NBO 6.0 software coupled to the Gaussian 09 rev. D.01 version. Then, the RunEDDB code (version 26-Jun-2021) was used to perform the EDDB analysis at the B3LYP/6-311++G**∼LANL2DZ//ZORA-BLYP-D3(BJ)/TZ2P level of theory.^97^ For the visualization of EDDB_G_(**r**) surfaces, we used the Avogadro1.0 molecular editor.^98^

Third, the aromatic stabilization energy was determined through homodesmotic reactions.^99,100^ Finally, steric effects were analyzed with the SambVca2.1 package of Cavallo and co-workers, calculating the %V_Bur_ and depicting steric maps.^101−103^

## 3 Results and Discussion

This section begins with the experimental considerations based on ^11^B{^1^H} NMR spectra of a series of iodinate derivatives that result from the stepwise substitution of hydrogen atoms with iodine atoms in the neutral *o*-carborane (1,2-C_2_B_10_H_12_). Then, the chemical oxidation of halogenated derivatives of icosahedral anionic boranes ([B_12_X_12_]^2–^, X = F, Cl, Br, I, with H as the reference) is presented. Subsequently, the discussion delves into the obtained computational results.

### 3.1 Initial Considerations

The benzene ring is possibly the ideal core to generate an additional ring to produce a concentric and coplanar two-ring system in which the second ring may result in an aromatic ring. Moreover, iodine is easily polarizable, is large, and can be oxidized. Hence, C_6_I_6_ was the best starting point to build a system with two aromatic rings orthogonal to each other. A perfect structure for double 3D-aromaticity would be a 3D-system whose core is an icosahedron, ideally the periodinated “B_12_”, [B_12_I_12_]^2–^, which could give rise to B_12_I_12_ by oxidation. A similar core structure would be that of the “C_2_B_10_”, i.e., periodinate C_2_B_10_I_12_.

**Figure 1:**
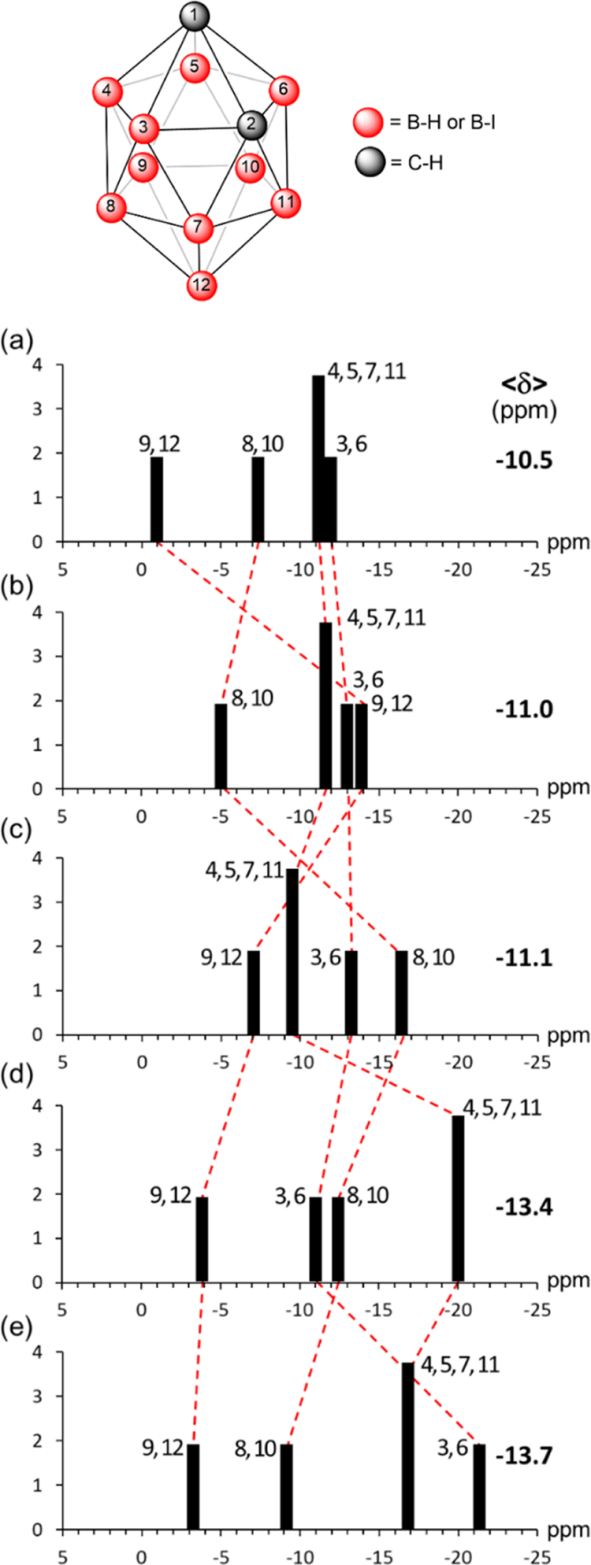
Schematic representation of the ^11^B NMR spectra from samples in acetone-d_6_ solutions with the peak assignments for unsubstituted *o*-carborane (a)^121^ and some iodinated derivatives: 9,12-I_2_-*o*-carborane (b),^110^ 8,9,10,12-I_4_-*o*-carborane (c),^122^ 4,5,7,8,9,10,11,12-I_8_-*o*-carborane (d),^123^ and 3,4,5,6,7,8,9,10,11,12-I_10_-*o*-carborane (e).^113^ The peak assignment was unambiguously done by means of a two-dimensional ^11^B{^1^H}–^11^B{^1^H} COSY NMR spectrum. On the right is shown the mean ^11^B{^1^H} NMR chemical shift for each compound. Figure drawn using the data of ref (124).

To understand the electronic structure and the influence of iodine substitution, one may consider the study of the evolution of [B_12_H_12_]^2–^ through the incorporation of iodine groups on the core cluster, step by step. Nevertheless, the step-by-step synthesis of these iodinated compounds cannot be achieved experimentally using the icosahedral cluster [B_12_H_12_]^2–^. However, it is feasible to accomplish this synthesis with the *o*-carborane cluster, as performed by some of us. The presence of two adjacent carbon atoms in the *o*-carborane cluster leads to variations in the charge density among its different vertices.^104^ As a result, it is experimentally feasible to obtain and isolate the step-by-step iodinated compounds derived from *o*-carborane while modulating both the number and the position of iodine atoms that replace the hydrogen atoms in the *o*-carborane cluster. The chemistry of mono- to ten-substituted boron-iodinated *o*-carboranes (I_n_-1,2-C_2_B_10_H_12-*n*_, *n* = 1–10) has been widely developed because the usefulness of *o-*carborane units is dependent upon their functionalization. The 2000s were dominated by the catalyzed reactions under Kumada conditions, and palladium was the sought catalyst for the B–C bond formation from B–I vertices, but more available metals (Ni) as well as the presence of CuI as cocatalyst were also employed to obtain well-defined patterns of *o*-carborane substitution from different and well-defined iodo-*o*-carboranes. Conventional reactions in solution as well as new solvent-free methods permitted regioselective control of *o-*carboranes iodine substitution to generate iodine I*_n_*-1,2-C_2_B_10_H_12-*n*_ (*n* = 1–10) derivatives on demand. The synthesis of regioselective B-iodinated *o*-carboranes was achieved; nevertheless, in general, the reactivity of the halogen linked to the boron is low. The ^11^B{^1^H} NMR spectra of the *o*-carboranes derivatives were used as a probe to analyze the changes in the electron distribution of *o*-carboranes due to substitution.^105−109^ Focusing on the ^11^B{^1^H} NMR spectrum of 9,12-I_2_-*o*-carborane (Figure 1b) and comparing it with *o*-carborane (Figure 1a), it can be observed that its appearance is considerably modified by the presence of iodine atoms on the B(9) and B(12) vertices with respect to pristine *o*-carborane and that the chemical shifts of these boron atoms are especially affected, being largely shifted upfield (from −2.1 ppm in the parent *o*-carborane to −13.8 ppm in the diiodinated *o-*carborane derivative, which corresponds to a Δδ = 11.7 ppm).^110^ The explanation that the substitution of hydrogen by iodine causes significant shielding on boron atoms attached to the substituent is that lone pairs of iodine interact efficiently with the *p* orbitals of *ipso* B, which produces a π backdonation from the I atom to the B atom of the substituted vertices. This enriches considerably the electron density at this B vertex, increasing its shielding and consequently shifting its B resonance to a high field.

Important is the fact that the pattern of the ^11^B{^1^H} NMR spectra of the parent *o*-carborane and the periodinated *o*-carborane from high to low frequency is the same 2:2:4:2, but the mean chemical shift value (⟨δ⟩) moves slightly upfield from −10.5 to −13.7 ppm as B-iodination of *o*-carborane moves from 0 to 10 atoms, respectively (Figure 1). These results suggest that by increasing the number of I atoms, the π backdonation ability of I atoms decreases, consistent with the formation of intramolecular I···I noncovalent bonding. In other words, the lone pairs of electrons on I are used in backdonation when there is no chance to interact with other neighboring iodine atoms, but when this possibility exists, there is a preference for noncovalent interactions. As a result, these electrons would be less prone for other endeavors, for instance, to be removed (oxidation) from the cluster. To evidence the presence of noncovalent intramolecular interactions in the boron periodinated I_10_-*o*-carborane, a search at the Cambridge Structural Database (CSD)^111,112^ was done. The CSD showed just three hits (CEHWOC, WUNDAL, and WUNDOZ). CEHWOC^113^ and WUNDAL^114^ have solvents as adducts in the crystal cells, but WUNDOZ was obtained solvent-free.^114^ To avoid any interference in measuring the I···I distances, these were measured on the WUNDOZ, giving values in the range 3.946–4.278 Å (the van der Waals radius of the I atom is 1.98 Å,^115^Figure S2) that parallel with the theoretical distances reported by the C_6_H_5_I···IC_6_H_5_ homodimers via type I–IV halogenhalogen interaction (3.96–4.15 Å).^116^ The boron periodinated I_10_-*o*-carborane crystal structure (WUNDOZ) supports the I···I noncovalent bonding formation and the creation of a two adjacent vertices truncated icosahedral (the two adjacent C–H vertices) constituted by the ten connected I atoms.

A similar situation is expected for the periodinated [B_12_I_12_]^2–^ that is even a better core than *o*-carborane to form a clustered onion-like structure. Removal of two electrons would seemingly lead to a clustered onion-like structure, but is this what occurs experimentally? When comparing the ^11^B{^1^H} NMR spectra in acetone of the parent Na_2_[B_12_H_12_] and its iodinated Na_2_[B_12_I_12_], it is seen that both display a singlet at the chemical shifts −15.4 (^1^J(B,H) = 125 Hz)^48,117^ and −15.8 ppm for Na_2_[B_12_H_12_] and Na_2_[B_12_I_12_], respectively. This NMR data is consistent with the data obtained for the *o*-carborane, to say that, as in the [B_12_H_12_]^2–^, the [B_12_I_12_]^2–^ cluster does not generate π backdonation to the *ipso* B atom, but most possibly, the iodine atoms are involved in intramolecular I···I noncovalent bonding interactions. Again, we moved to the CSD to confirm this hypothesis.^111,112^ CSD showed just 16 hits for salts of [B_12_I_12_]^2–^, but only two hits (QAVVAO and ASIWAC)^118^ were found for its sodium salts. QAVVAO corresponds to a crystal containing Na_2_[B_12_I_12_], DMF, and γ-cyclodextrin with low-resolution reflections, and ASIWAC corresponds to Na_2_[B_12_I_12_]·8SO_2_ with good resolution. The I···I distances in the ASIWAC crystal structure were measured in the 3.982–4.112 Å range, which is consistent with the presence of noncovalent I···I bonds between the 12 iodine atoms bonded *exo*-cluster to the B_12_ icosahedron. All of these results support the formation of two concentric layers in a clustered onion-like structure, one made by 12 B atoms connected by multicenter bonds (the inner)^119^ and 12 iodine atoms connected by noncovalently bonded halogen bonds (the outer).

If we consider the oxidation of periodobenzene C_6_I_6_, two methods were utilized. In one method, chlorine is bubbled into a suspension of orange C_6_I_6_ in a mixture of triflic acid and trifluoroacetyl triflate (TFAT). In this process, besides [C_6_I_6_]^2+^, the radical [C_6_ClI_5_]^·+^ was obtained. In the same report, an alternative method to produce [C_6_I_6_]^2+^ involves the use of pertriflic acid (CF_3_SO_4_H) or H_2_O_2_ in triflic acid as the oxidizing agent. The mass spectrum displays peaks at *m*/*z* 834 corresponding to [C_6_I_6_]^+^ and at *m*/*z* 417 corresponding to [C_6_I_6_]^2+^. Remarkable was the ^13^C{^1^H} NMR spectrum that produced a unique ^13^C resonance, indicating the equivalence of all C atoms and, indeed, that the compound was diamagnetic.^15^

If we now examine the 3D-aromatic core per excellence, the [B_12_H_12_]^2–^ and its perhalogenated dianions [B_12_X_12_]^2–^ (X= F, Cl, Br, I), it is seen that in liquid sulfur dioxide, they can be oxidized either by chemical or electrochemical methods to give stable radicals.^73^ The chemical oxidation is done with the strong oxidizing agent AsF_5_ that yields the radical anions [B_12_X_12_]^·–^ (X = F, Cl, Br), but it fails to produce [B_12_I_12_]^·–^. When an excess of AsF_5_ was used, the neutral *hypercloso*-boranes B_12_X_12_ (X = Cl, Br) were obtained but failed to produce B_12_I_12_. The explanation given was the weakness of the B–I bond that would agree with the lack of π backdonation argued above and consistent with the stepwise loss of I^·^ radicals leading to B_12_ demonstrated by electrospray ionization-trap mass spectrometry.^120^ B_12_Cl_12_ and B_12_Br_12_ were characterized by X-ray diffraction (XRD) and did show unequal B–B bonds characteristic of *hypercloso* species.^73^ Electrochemical oxidation of [B_12_X_12_]^2–^ (X = F, Cl, Br, I) shows that the first and second oxidation processes are detected for X = F, Cl, Br but not for X = I. Remarkable is the high oxidation potentials required for the oxidation, in the range of +1.68 and +2.29 V vs Fc^1+/0^ for the first process that is quasi reversible while the second process, in the range of +2.63 to +2.71 V vs Fc^1+/0^, is irreversible.

### 3.2 Molecular Structure

**Figure 2:**
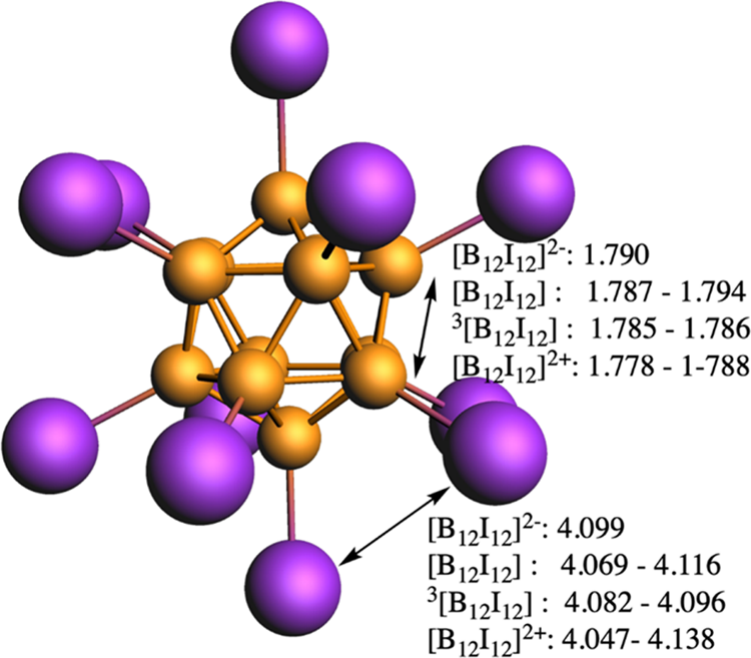
B–B and I···I bond distances range (in Å) of closed-shell singlet [B_12_I_12_]^2-/0/2+^ and triplet ^3^[B_12_I_12_].

First, we quantum chemically studied the singlet ground state of [B_12_I_12_]^2–^ as a possible species with double 3D-aromaticity, the borane cage following the 2*N* + 2 Wade rule with *N* = 12, and the I_12_ sphere with 72 electrons as lone pairs following the Hirsch rule. As said before, to reach double aromaticity, a double oxidation may be required to open a hole in one of the 5p orbitals of iodine. Therefore, we analyzed [B_12_I_12_] and also [B_12_I_12_]^2+^. The first gas-phase ionization Gibbs energy of [B_12_I_12_]^2–^ to [B_12_I_12_]^·–^ is 35.2 kcal/mol, and the second ionization to produce singlet [B_12_I_12_] requires 101.3 kcal/mol. It has to be said that the triplet state of [B_12_I_12_] is the ground state, and it is more stable than the closed-shell and the open-shell singlet states by 5.7 and 3.7 kcal/mol, respectively. Further oxidation to singlet [B_12_I_12_]^2+^ costs 408.2 kcal/mol.^125^ The ground state of [B_12_I_12_]^2+^ is a quintet that is more stable than the closed-shell singlet state by 6.9 kcal/mol. The molecular structure of [B_12_I_12_]^2–^ has *I*_*h*_ symmetry with B–B bond length of 1.790 Å (see Figure 2) and, therefore, the bond length alternation (BLA), i.e., the difference between the shortest and the longest B–B distance, is zero. Our optimized geometries indicate that the shortest I···I distance in [B_12_I_12_]^2–^ is 4.099 Å, 0.539 Å longer than that of C_6_I_6_ (3.560 Å). Despite the relatively long I···I distance in [B_12_I_12_]^2–^, there is almost no space left in the I_12_ crown with a %V_Bur_ = 91.4% in comparison to [B_12_H_12_]^2–^ for which %V_Bur_ = 41.7% (Figures S23–S26). Double oxidation removes two electrons from the triply degenerated HOMO orbital of [B_12_I_12_]^2–^, which is I···I antibonding (Figure S1). As a consequence, the I···I distance is reduced minutely to 4.082 Å (triplet) or to 4.069 Å (closed-shell singlet), and the B–B bond lengths become also somewhat shorter, ranging from 1.785 to 1.786 Å (triplet) or 1.787 to 1.794 Å (singlet) with a BLA of 0.001 Å for the triplet and 0.007 Å for the singlet. The two electrons are removed from the I_12_ shell, which gets a positive charge of 0.410 (triplet) or 0.408 (singlet) electrons according to Voronoi deformation density (VDD) charges,^126^ computed at the ZORA-BLYP-D3(BJ)/TZ2P level of theory. Finally, oxidation to closed-shell singlet [B_12_I_12_]^2+^ leads to a minor reduction of the B–B and I···I distances (BLA being 0.010 Å). Again, the electrons are removed from the I_12_ shell, which becomes positive by 2.512 electrons (using Hirshfeld charges,^127^ we got 0.609 and 2.657 e for the I_12_ unit of ^3^B_12_I_12_ and B_12_I_12_^2+^, respectively). The number of resonance structures that can be built in the I_12_ shell is 12 for B_12_I_12_ if the doubly positive charge on I_12_ is located in a single I atom and 66 if the two positive charges are placed in two different I atoms. For [B_12_I_12_]^2+^, we have 66 resonance structures if the two doubly positive charges are located in two I atoms and 495 resonance structures if the positive charges are placed in four different I atoms. In both cases, the boron core remains with 26 electrons.

### 3.3 Magnetic Aspects

**Figure 3:**
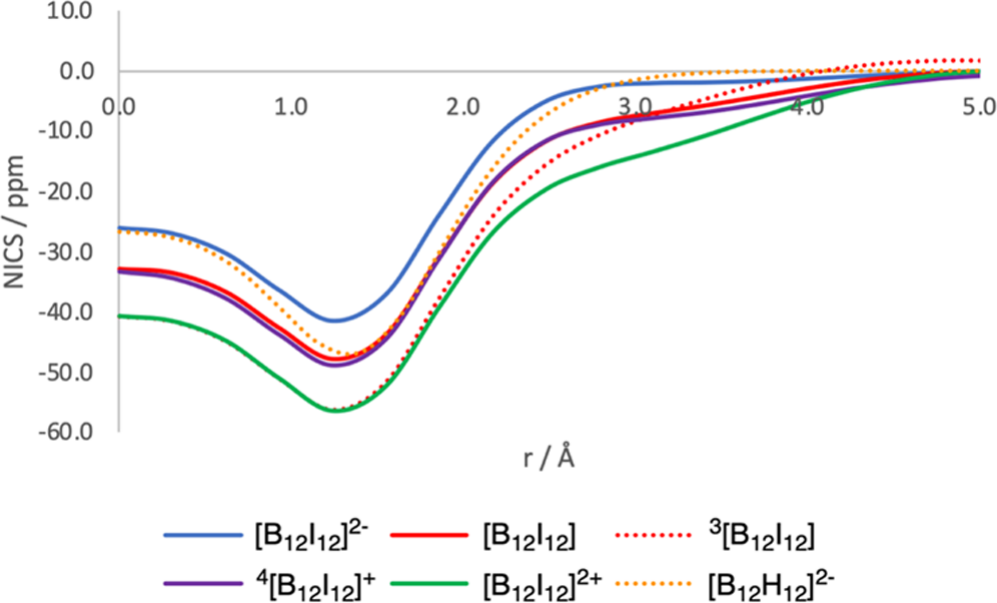
NICS scan (ppm) from the center of the boron cluster to the middle of the closest I_3_ (or H_3_) three-membered ring for [B_12_I_12_]^2–^ (singlet), [B_12_I_12_] (singlet and triplet), [B_12_I_12_]^+^ (quartet), and [B_12_I_12_]^2+^ (singlet) clusters. Comparison to [B_12_H_12_]^2–^ (singlet) is included. Distances in Å.

To determine the magnetic behavior of our systems, we first performed NICS scans moving from the center of the dodecahedron to the center of the closest X_3_ (X = H, I) three-membered ring (3-MR). As seen in Figure 3, the NICS scans for [B_12_I_12_]^2–^ and [B_12_H_12_]^2–^ clusters point out a somewhat higher aromaticity for [B_12_H_12_]^2–^. The minimum of the NICS scan in all cases is found around the B_3_ plane. NICS scans of [B_12_I_12_]^2–^ and [B_12_H_12_]^2–^ reflect the 3D-aromaticity of the borane cage and the absence of further aromaticity around the X_12_ sphere (X = H, I). When we compare the NICS scans of [B_12_I_12_]^2–^ and [B_12_I_12_] in their closed-shell singlet and triplet states, the latter show significantly more negative values of about 10 ppm in the closed-shell singlet and 20 ppm in the triplet ground state than [B_12_I_12_]^2–^ (for the open-shell singlet, the difference is even higher, of ca. 30 ppm, Figure S3), which is an indication of the presence of magnetic ring currents in the I_12_ shell in closed- and open-shell singlet and triplet states of B_12_I_12_ despite the fact that now the I_12_ shell has 70 electrons (35 electron pairs) and, consequently, does not follow the Hirsch rule for spherical aromaticity. In contrast, [B_12_H_12_] is antiaromatic, as the two electrons are removed from the borane cage (Figure S4). Subsequent oxidation of [B_12_I_12_] to yield closed-shell singlet [B_12_I_12_]^2+^ produces a further decrease of the NICS values, although differences between closed-shell singlet [B_12_I_12_] and [B_12_I_12_]^2+^ are now smaller (for the triplet and quintet [B_12_I_12_]^2+^, the aromaticity is increased in the former and reduced in the latter, Figures S5 and S6). NICS scans of C_6_H_6_, C_6_I_6_, and [C_6_I_6_]^2+^ follow a trend similar to that found for [B_12_H_12_]^2–^, [B_12_I_12_]^2–^, and [B_12_I_12_] (Figure S7). Based on NICS values, one may therefore consider [B_12_I_12_] and [B_12_I_12_]^2+^ as doubly 3D-aromatic.

We have also analyzed the quartet state of [B_12_I_12_]^+^ because, in this state, the three orbitals of the triply degenerated HOMO are occupied with a single same spin electron, leading to an *I*_*h*_ symmetric structure with bond length equalization. So, we expect this state to be particularly doubly aromatic since it reminds the electronic distribution of Baird aromatic species.^38^ However, according to the NICS scans of Figure 3, this state has an aromaticity slightly smaller than that of closed-shell singlet [B_12_I_12_]^2+^. Other systems with double 3D-aromaticity according to NICS values (Figures S8 and S12) are [B_12_Br_12_]^0/2+^ (d_Br–Br_ = 4.069/4.047 Å), [B_6_I_6_]^2+^ (d_I···I_ = 4.712 Å), and [B_14_I_14_]^0/2+^ (longest d_I···I_ = 4.185/4.210 Å) but not [B_10_I_10_]^0/2+^ (longest d_I···I_ = 4.693/4.699 Å). The case of B_12_Br_12_ is important because (i) it shows that efficient overlap between 4p orbitals of Br atoms is possible, and (ii) this species has been detected in the oxidation process of [B_12_Br_12_]^2–^.^73^

**Figure 4:**
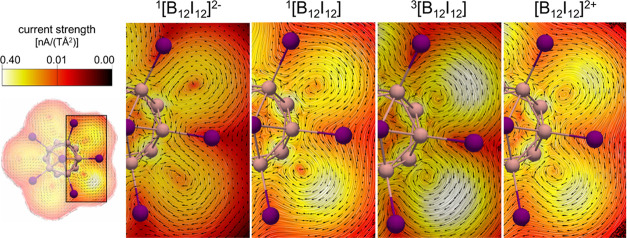
Current-density susceptibility in the magnified area (black rectangle) of the ^1^[B_12_I_12_]^2–^, singlet and triplet [B_12_I_12_], and 1[B_12_I_12_]^2+^ computed in a plane at 0.8 Å (see Figure S13). The color scale corresponds to the strength of the modulus of the current-density susceptibility in the range 0.0001 (dark red) to 0.4 (white) nA/(TÅ^2^).

To confirm that the results of the NICS scan stem from diatropic ring currents, we computed the current-density susceptibility and visualized the current at the plane located in the middle of the two boron 5-MRs (0.0 Å), the plane including one of the boron 5-MR (0.8 Å), and the plane of iodine 5-MR (1.8 Å) for [B_12_I_12_]^2–^ for the singlet and triplet states of [B_12_I_12_] and [B_12_I_12_]^2+^, as well as for [B_12_H_12_], C_6_I_6_, and [C_6_I_6_]^2+^ (Figures S13–S16). As can be seen, the changes in the current-density susceptibility around the iodine substituents are similar on going from C_6_I_6_ to [C_6_I_6_]^2+^ and from [B_12_I_12_]^2–^ to [B_12_I_12_]. This further substantiates the potential double 3D-aromaticity in [B_12_I_12_] and [B_12_I_12_]^2+^ when evaluated with magnetic aromaticity descriptors. Figure 4 shows the current-density susceptibility of [B_12_I_12_]^2–^, singlet and triplet [B_12_I_12_], and [B_12_I_12_]^2+^ in the 0.8 Å plane, which is ∼1 Å above one of the I 5-MR planes. In this plane, differences between the differently oxidized clusters are more evident. From these pictures, one can see the generation of a current density around the iodine substituents when going from [B_12_I_12_]^2–^ to [B_12_I_12_] and [B_12_I_12_]^2+^. This current is particularly intense for ^3^[B_12_I_12_]. In addition, we have computed the ring currents for [C_2_B_10_I_12_] and [C_2_B_10_I_12_]^2+^ in their singlet and triplet states (Figures S17 and S18), which also show an increase in the current-density around the iodine substituents after oxidation.

### 3.4 Electronic Aspects

**Figure 5:**
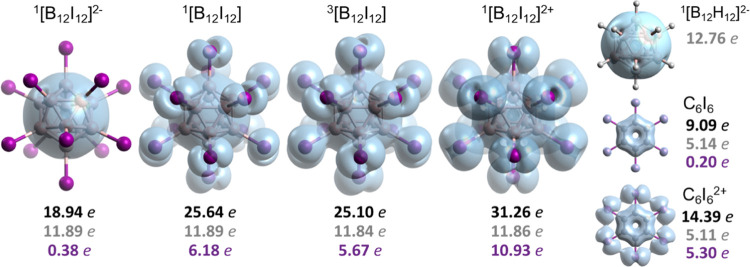
Isosurfaces (isocontour 0.007 e) of the electron density of delocalized bonds (EDDB) for [B_12_I_12_]^2–^, [B_12_I_12_] singlet and triplet states, and [B_12_I_12_]^2+^, as well as [B_12_H_12_]^2–^, C_6_I_6_, and [C_6_I_6_]^2+^ for comparison purposes. Numerical results correspond to the EDDB_G_ population of the whole system (black), boron/carbon atoms (gray), and iodine (purple) atoms separately.

Yet, other aspects of the potential double 3D-aromaticity (electronic, energetic, and geometric) must also be assessed, and next, we have analyzed the electron delocalization using the electron density of delocalized bonds (EDDB) method. The EDDB method decomposes the one-electron density in several “layers” corresponding to different levels of electron delocalization,^128^ namely, the density of electrons localized on atoms (EDLA) representing inner shells, lone pairs, etc.; the electron density of localized bonds (EDLB) representing typical (2-center 2-electron) Lewis-like bonds; and EDDB, which represents electron density that cannot be assigned to atoms or bonds due to its (multicenter) delocalized nature. The EDDB population of electrons delocalized in a 2D- or 3D-closed circuit can be used as an indicator of aromaticity.^129^ According to the EDDB, the number of delocalized electrons (or holes) increases upon oxidation of [B_12_I_12_]^2–^ (Figure 5). The number of delocalized electrons in the borane cage remains more or less similar, while the electron delocalization of the iodine sphere increases from 0.38 e ([B_12_I_12_]^2–^) to 6.18 e (singlet [B_12_I_12_]), 5.67 e (triplet [B_12_I_12_]), and to 10.93 e ([B_12_I_12_]^2+^). In the case of C_6_I_6_, we also have the delocalization around the I_6_ circle going from 0.20 e in C_6_I_6_ to 5.30 e in [C_6_I_6_]^2+^. For [B_12_I_12_]^2+^, the number of delocalized electrons in the iodine shell (10.93 e out of 48 e coming from the 24 electron pairs located in the spherical surface of I_12_) is similar to that of the borane cage (11.86 e out of 26 e). However, the percentage of delocalized electrons is still much larger for the borane cage (46 vs 23%). Moreover, the EDDB surface does not cover the entire I_12_ spherical surface. As a whole, the EDDB shows that there is electron delocalization among the I_12_ atoms (as expected from the different resonance structures) but does not fully support the existence of double 3D-aromaticity in [B_12_I_12_] and [B_12_I_12_]^2+^, especially in the former. EDDB plots for [C_2_B_10_I_12_] and [C_2_B_10_I_12_]^2+^ in their singlet and triplet states show very similar behavior (Figure S19), the electron delocalization being clearly higher for [C_2_B_10_I_12_]^2+^ than for [C_2_B_10_I_12_], especially in its singlet state.

### 3.5 Energetic Aspects and the Nature of the I···I Chemical Bond

As discussed before, the relatively short I···I distances in 3,4,5,6,7,8,9,10,11,12-I_10_-*o*-carborane support the existence of noncovalent I···I bonding. We expect a similar situation in [B_12_I_12_]^2–^. Moreover, we know that the I···I distance decreases when we move from [B_12_I_12_]^2–^ to [B_12_I_12_]^0/2+^. Do we reach a situation that can be defined as covalent I–I bonding in [B_12_I_12_]^0/2+^? To answer this question, we performed a quantum theory of atoms-in-molecules (QTAIM) analysis for these species, and the results show that none of them has a bond critical point (BCP) between the I atoms, thus unambiguously indicating that we have noncovalent I···I interaction but not covalent bonding. On the other hand, in the case of [C_6_I_6_]^2+^, we observe I···I BCPs (Figure S20), although the density at these BCPs is very low (ρ_BCP,I···I_ = 0.019 au), thus precluding the existence of covalent bonding. The highest overlap between lone pair orbitals in two adjacent I atoms in C_6_I_6_ and [B_12_I_12_]^2–^ is 0.165 and 0.097 au, respectively.

Thus, a crucial question is if it is possible to have aromaticity without covalent bonding. The answer from magnetic and electronic indices seems to be yes for [C_6_I_6_]^2+^ and maybe for [B_12_I_12_]^0/2+^. Yet, it is important to emphasize that aromaticity is linked to stability, and we already pointed out that the [B_12_I_12_]^0/2+^ compounds have not been isolated. To check whether these molecules are stabilized by the double 3D-aromaticity, we designed several homodesmotic^83^ reactions (Tables 1 and S2). We have chosen the position of the H atoms so that in [B_12_I_6_H_6_]^0/2+^ or in [C_6_H_3_I_3_]^2+^, the potential double aromaticity is quenched (for instance, in [C_6_H_3_I_3_]^2+^, the three H atoms are located in *meta* positions with respect to each other). As can be seen from reaction (3) in Table 1, there is a stabilization of [C_6_I_6_]^2+^ because of the double 2D-aromaticity. In contrast, in the case of [B_12_I_12_]^0/2+^, such stabilization does not take place (reactions (1) and (2)). Therefore, the energetic indicator of aromaticity does not support the double 3D-aromaticity of [B_12_I_12_]^0/2+^, but it reinforces the conclusion that [C_6_I_6_]^2+^ is doubly 2D-aromatic. So, the answer to the question of whether it is possible to have aromaticity without covalent bonding is that, in some particular cases such as in [C_6_I_6_]^2+^, it seems possible. Lack of covalent I···I bonding in [C_6_I_6_]^2+^ is substantiated by the low density of I···I BCPs, by the population of molecular orbitals with antibonding I···I character, and the large distance between I atoms far outside the sum of the covalent radii of two I atoms (r_I_^cov^ = 1.39 Å).^130^ In this context, let us mention the synthesized dismutational aromatic isomer of hexa-silabenzene (Si_6_R_6_),^131^ which shows strong diamagnetic response when exposed to an external magnetic field and has two unsubstituted silicon atoms with an interatomic distance that is significantly longer than the common covalent Si–Si single bond.^132^

**Table 1:** Electronic and Gibbs Reaction Energies (in kcal/mol) of Selected Homodesmotic Reactions^a^

**reaction**	**Δ***E*	**Δ***G*
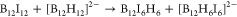 1	–37.5	–40.1
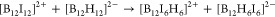 2	–2.8	–6.5
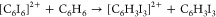 3	37.1	34.1

a All species computed in their closed-shell singlet state. In the case of reaction (1), if B_12_I_12_ and B_12_I_6_H_6_ are in a triplet state, Δ*E* = −37.9, and Δ*G* = −40.7 kcal mol^–1^.

Furthermore, one can note the pseudo-π method of Steiner and Fowler that replaces CH units in conjugated hydrocarbons by H atoms with a single 1*s* function located at the original C positions.^133^ In that way, they reproduce the ring currents of π-conjugated hydrocarbons at a low cost. However, in the pseudo-π model of benzene, there is no H···H covalent bonding or only a very weak one^134^ (the H atoms are placed far from each other and ρ_BCP,H···H_ = 0.056 au, whereas for the H_2_ molecule, the ρ_BCP,H–H_ is 0.266 au). Moreover, the energy of the cyclic H_6_ system is higher than those of the three H_2_ molecules by 128.5 kcal/mol. So, there is no aromaticity in these H cycles (lack of stabilization), but there are ring currents and extensive electron delocalization (EDDB_G_ indicates that 5.43 e out of a total of 6 are delocalized, Figure S27). This situation is akin to that found for [B_12_I_12_]^0/2+^.

Finally, a lack of stability of B_12_I_12_ explains why [B_12_I_12_]^2–^ requires such high oxidation potentials or strong oxidizing agents to reach the first oxidation step, the monoanionic radical, and even higher potentials to produce the neutral species.

## 4 Conclusions

^11^B NMR spectra of I_n_-*o*-C_2_B_10_H_12-n_ are consistent with the existence of intramolecular noncovalent I···I bonding when *n* is large and, in particular, in I_10_-*o*-C_2_B_10_H_2_. It is expected that such a bonding situation is also present in [B_12_I_12_]^2–^. Based on the example of double 2D-aromaticity in [C_6_I_6_]^2+^, we analyzed the possibility of double 3D-aromaticity in [B_12_I_12_] and [B_12_I_12_]^2+^. Calculations of magnetic and electronic descriptors of aromaticity suggest (the electronic to less extent) that these two oxidized forms of *closo* icosahedral dodecaiodo-dodecaborate cluster, [B_12_I_12_] and [B_12_I_12_]^2+^, can be classified as doubly 3D-aromatic compounds, much like [C_6_I_6_]^2+^ is considered doubly 2D-aromatic. However, consistent with experimentation, homodesmotic reactions rule out the existence of double 3D-aromaticity in [B_12_I_12_]^0/2+^ because these reactions are all endothermic and endergonic for these species. Aromaticity is a complex phenomenon; aromatic compounds have many interesting properties, but two are essential, namely, *electron delocalization and energetic stabilization*. A lack of one of these two effects precludes the existence of aromaticity. Although the two species display noticeable magnetic ring currents and electron delocalization (as expected from the multiple existing resonance structures with the same weight), we cannot conclude that there is aromaticity on the I_12_ shell of [B_12_I_12_]^0/2+^ because homodesmotic reactions show that there is *no resonance stabilization*, one of the two main conditions required for aromaticity. These systems are good examples illustrating that analyses of aromaticity have to be exhaustive and are not focused only on one of the aspects (usually magnetic properties) that characterize aromatic compounds. [B_12_I_12_]^0/2+^ shows that diatropic ring currents and bond length equalization, leading to high symmetry, are important but not sufficient conditions for claiming aromaticity.

Finally, let us mention that the difficulty of generating B_12_I_12_ with electrochemical procedures already foreshadowed its lack of double 3D-aromaticity. Aromaticity, which is associated with electron delocalization facilitated by efficient orbital overlap, generally assists the synthesis of aromatic compounds because of their increased stability. However, in the case of B_12_I_12_, inefficient orbital overlap avoids the formation of a stable compound. This case highlights the potential for alternative approaches to enhancing stability beyond aromaticity. One such possibility is the generation of multiple weak, noncovalent I···I contacts, which, when combined, significantly boost stability.

## Supporting Information Available

A detailed description of the computational method, GIMIC calculation parameters used, additional homodesmotic reactions, additional molecular structures and QTAIM analysis, Cartesian coordinates of all studied species, HOMO and LUMO orbitals of [B_12_I_12_]^2–^, together with additional NICS scans, current-density plots, and steric maps (PDF)
