# Correction to “Single—Not Double—3D-Aromaticity in an Oxidized *Closo* Icosahedral Dodecaiodo-Dodecaborate Cluster”

**DOI:** 10.1021/jacs.4c17897

**Published:** 2025-01-09

**Authors:** Jordi Poater, Sílvia Escayola, Albert Poater, Francesc Teixidor, Henrik Ottosson, Clara Viñas, Miquel Solà

To compute the aromatic stabilization energy (ASE) of the double aromatic [C_6_I_6_]^2+^ species, we proposed the homodesmotic reaction shown in eq 1:
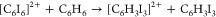
1The [C_6_I_6_]^2+^ species is doubly aromatic, whereas the three other species display single aromaticity; therefore, the energy difference between the products and reactants of eq 1 should provide an estimate of the ASE due to the aromaticity of the I_6_ ring in [C_6_I_6_]^2+^.

We recently realized that [C_6_H_3_I_3_]^2+^ is not aromatic because, in the oxidation process of C_6_H_3_I_3_, the electrons are removed from the π-system and not from the iodine atoms. The reason is the following. The HOMO of C_6_I_6_ corresponds to the most antibonding orbital formed by the in-plane *5p* orbitals of the iodine atoms. This HOMO energy is stabilized in C_6_H_3_I_3_ because the overlap between the *1s* orbitals of H atoms and the *5p* orbitals of I atoms is reduced, and, for this system, the HOMO corresponds to the π-system. Because of that, the ASE of the I_6_ ring in [C_6_I_6_]^2+^ (Δ*E* = 37.1 kcal/mol and Δ*G* = 34.1 kcal/mol) is overestimated by eq 1. A more correct estimation of this ASE can be obtained from eq 2:
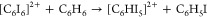
2In this equation, the oxidation of C_6_HI_5_ removes the electrons from the I atoms; consequently, [C_6_HI_5_]^2+^ remains π-aromatic. The ASE obtained from eq 2 is Δ*E* = 12.6 kcal/mol and Δ*G* = 11.7 kcal/mol at the ZORA-BLYP-D3(BJ)/TZ2P level of theory.

The significant reduction in the value of the ASE of the I_6_ ring in [C_6_I_6_]^2+^ by replacing eq 1 with eq 2 does not change the main conclusions of the work. This Correction highlights the importance of thoroughly scrutinizing conclusions on (anti)aromaticity effects, as the various (anti)aromaticity descriptors have different pitfalls that are often not sufficiently discussed in the research community.

