# Supplementary material for: Single—Not Double—3D-Aromaticity in an Oxidized Closo Icosahedral Dodecaiodo-Dodecaborate Cluster
Source: J Am Chem Soc. 2023 Sep 20;145(41):22527–38. doi: 10.1021/jacs.3c07335 (PMC10591335; doi:10.1021/jacs.3c07335)
Supplement: Supplementary file 1 — ja3c07335_si_001.pdf [file ja3c07335_si_001.pdf]

SUPPORTING INFORMATION

Single – not double – 3D-aromaticity in oxidized *closo* icosahedral dodecaiodo-dodecaborate cluster

Jordi Poater,[a,b§] Sílvia Escayola,[c,d§] Albert Poater,[c] Francesc Teixidor,[e] Henrik Ottosson*,[f] Clara Viñas*,[e] and Miquel Solà[c]*

[a] *Departament de Química Inorgànica i Orgànica & IQTCUB, Universitat de Barcelona, Martí i Franquès 1-11, 08028 Barcelona, Spain.*

[b] *ICREA, Pg. Lluís Companys 23, 08010 Barcelona, Spain.*

[c] *Institut de Química Computacional i Catàlisi, Departament de Química, Universitat de Girona, C/ Maria Aurèlia Capmany, 69, 17003 Girona, Catalonia, Spain.*

[d] *Donostia International Physics Center (DIPC), 20018 Donostia, Euskadi, Spain.*

[e] *Institut de Ciència de Materials de Barcelona, Consejo Superior de Investigaciones Científicas, Campus Universitat Autònoma de Barcelona, 08193 Bellaterra, Spain.*

[f] *Department of Chemistry - Ångström Laboratory, Uppsala University, 751 20 Uppsala, Sweden.*

Table of Contents

Figure S1. Frontier HOMO and LUMO molecular orbitals of $[B_{12}I_{12}]^{2-}$. S4

Table S1. GIMIC calculation parameters. S5

Figure S2. Crystal structures displaying the I–I distances. a) The neutral I_{10}-o-carborane (WUNDOZ) containing DMS and acetone (CEHWOC);[1] which are in the range 3.946-4.278 Å. b) the neutral I_{10}-*o*-carborane[2] in which the distances are in the range 3.916-4.212 Å and, c) $Na_2[B_{12}I_{12}]\cdot 8SO_2$ (the Na^+ cation and SO_2 molecules omitted for clarity)[3] in which the distances I–I are in the range 3.982 – 4.112 Å. The van der Waals radii of the I atom is 1.98 Å.[4] S6

Figure S3. NICS scan (in ppm) from the center of the boron cluster to the middle of the closest I_3 three-membered ring for $[B_{12}I_{12}]$ cluster in singlet, open-shell singlet, and triplet states. Distances in Å. S7

Figure S4. NICS scan (in ppm) from the center of the boron cluster to the middle of the closest H_3 three-membered ring for $[B_{12}H_{12}]^{2-}$ and $[B_{12}H_{12}]$ clusters. Distances in Å. S7

Figure S5. NICS scan (in ppm) from the center of the boron cluster to the middle of the closest I_3 three-membered ring for $[B_{12}I_{12}]^{2+}$ cluster in singlet, triplet, and quintet states. Distances in Å. S8

Figure S6. NICS scan (in ppm) from the center of the boron cluster to the middle of the closest I_3 three-membered ring for $[B_{12}I_{12}]^{2-}$ (singlet), $[B_{12}I_{12}]$ (triplet), $[B_{12}I_{12}]^{+}$ (quartet), and $[B_{12}I_{12}]^{2+}$ (quintet) clusters. Distances in Å. S8

Figure S7. NICS scan (in ppm) perpendicular from the center of the ring for C_6I_6 and $[C_6I_6]^{2+}$ clusters. C_6H_6 has been added for comparison. Distances in Å. S9

Figure S8. Shortest I···I bond length (in Å) of the boron clusters under analysis. $C_6I_6^{2+}$ has also been enclosed for comparison. S10

Figure S9. NICS scan (in ppm) from the center of the boron cluster to the middle of the closest I_3 three-membered ring for $[B_6I_6]^{2-}$, $[B_6I_6]$, and $[B_6I_6]^{2+}$ clusters. Distances in Å. S11

Figure S10. NICS scan (in ppm) from the center of the boron cluster to the middle of the closest I_3 three-membered ring for $[B_{10}I_{10}]^{2-}$, $[B_{10}I_{10}]$, and $[B_{10}I_{10}]^{2+}$ clusters. Distances in Å. S11

Figure S11. NICS scan (in ppm) from the center of the boron cluster to the middle of the closest I_3 three-membered ring for $[B_{14}I_{14}]^{2-}$, $[B_{14}I_{14}]$, and $[B_{14}I_{14}]^{2+}$ clusters. Distances in Å. S12

Figure S12. NICS scan (in ppm) from the center of the boron cluster to the middle of the closest I_3 three-membered ring for $[B_{12}Br_{12}]^{2-}$, $[B_{12}Br_{12}]$, and $[B_{12}Br_{12}]^{2+}$ clusters. Distances in Å. S12

Figure S13. Orientation of the external magnetic field and perpendicular planes where the current density susceptibility has been sampled. S13

Figure S14. Current-density susceptibility of a) C_6I_6, and b) $C_6I_6^{2+}$ computed at the molecular plane, and the plane at 1 Å. The color scale corresponds to the strength of the modulus of the current-density susceptibility in the range of 0.0001 (red) to 0.4 (white) nA/T/Å^2. ....S13

Figure S15. Current-density susceptibility of $[B_{12}H_{12}]^{2-}$ in the planes represented in Figure 1. The color scale corresponds to the strength of the modulus of the current-density susceptibility in the range of 0.0001 (red) to 0.4 (white) nA/T/Å^2. ....S13

Figure S16. Current-density susceptibility of $[B_{12}I_{12}]^{2-}$, singlet and triplet $[B_{12}I_{12}]$, and $[B_{12}I_{12}]^{2+}$ in the planes represented in Figure 1. The color scale corresponds to the strength of the modulus of the current-density susceptibility in the range of 0.0001 (red) to 0.4 (white) nA/T/Å^2. ....S14

Figure S17. Orientation of the external magnetic field and perpendicular planes where the current density susceptibility has been sampled in the $^{X}[C_2B_{10}H_2I_{10}]^{N}$ species, where X = 1 or 3 and N = 0 or +2. ....S15

Figure S18. Current-density susceptibility of $[C_2B_{10}H_2I_{10}]$ and singlet and triplet $[C_2B_{10}H_2I_{10}]^{2+}$ in the planes represented in Figure S17. The color scale corresponds to the strength of the modulus of the current-density susceptibility in the range of 0.0001 (red) to 0.4 (white) nA/T/Å^2. ....S16

Figure S19. Isosurfaces (isocontour 0.007 e) of the electron density of delocalized bonds (EDDB). Numerical results correspond to $EDDB_G$ population of the whole system (black), boron and carbon (gray) and iodine (purple) atoms separately. S17

Figure S20. QTAIM analysis of $C_6I_6^{+2}$ and $B_{12}I_{12}$. S17

Figure S21. NICS scan (in ppm) from the center of the boron cluster to the middle of the closest I_3 three-membered ring for $[B_{12}I_{12}]^{2-}$ and singlet $[B_{12}I_{12}]$ clusters computed with the the ZORA-BLYP-D3(BJ)/TZ2P (solid line) and the B3LYP/6-311++G**~LANL2DZ (dotted line) methods. Distances in Å. S18

Figure S22. NICS scan (in ppm) from the center of the center of the benzene ring for B_6I_6 and singlet $[B_6I_6]^{2+}$ species computed with the the ZORA-BLYP-D3(BJ)/TZ2P (solid line) and the B3LYP/6-311++G**~LANL2DZ (dotted line) methods. Distances in Å. S19

Figure S23. %V_{Bur} and topographic steric maps in Å (XY plane) for $[B_{12}I_{12}]^{2-}$ using the center for each molecule or set of atoms under analysis, with a radius of 5.0 Å and with the Z axis defined by the 2 most opposed atoms of boron in $[B_{12}I_{12}]^{2-}$. The XY plane is perpendicular to the Z axis and contains the center of the molecule. S20

Figure S24. %V_{Bur} and topographic steric maps in Å (XY plane) for $[B_{12}Br_{12}]^{2-}$ using the center for each molecule or set of atoms under analysis, with a radius of 5.0 Å and with the Z axis defined by the 2 most opposed atoms of boron in $[B_{12}Br_{12}]^{2-}$. The XY plane is perpendicular to the Z axis and contains the center of the molecule. S21

Figure S25. %V_{Bur} and topographic steric maps in Å (XY plane) for $[B_{12}H_{12}]^{2-}$ using the center for each molecule or set of atoms under analysis, with a radius of 5.0 Å and with the Z axis defined by the 2 most opposed atoms of boron in $[B_{12}H_{12}]^{2-}$. The XY plane is perpendicular to the Z axis and contains the center of the molecule. S22

Figure S26. %V_{Bur} and topographic steric maps in Å (XY plane) using as centre the average of a set of 5 halides (or hydrogens) forming a plane as centre, with Z axis using the apical halide (or hydrogen) and any of the previous 5 halides (or hydrogens) to define the XZ axis for $[B_{12}I_{12}]^{2-}$, $[B_{12}Br_{12}]^{2-}$, and $[B_{12}H_{12}]^{2-}$..S23

Table S2. Electronic and Gibbs reaction energies (in kcal mol^{-1}) of selected homodesmotic reactions. S24

Figure S27. Isosurfaces (isocontour 0.007 e) of the electron density of delocalized bonds (EDDB) for H_6 model system...S24

Table S3. Cartesian coordinates and ADF total electronic energy (in parentheses and in kcal mol^{-1}) of $B_{12}I_{12}$ clusters under analysis computed at the ZORA-BLYP-D3(BJ)/TZ2P level of theory. S25

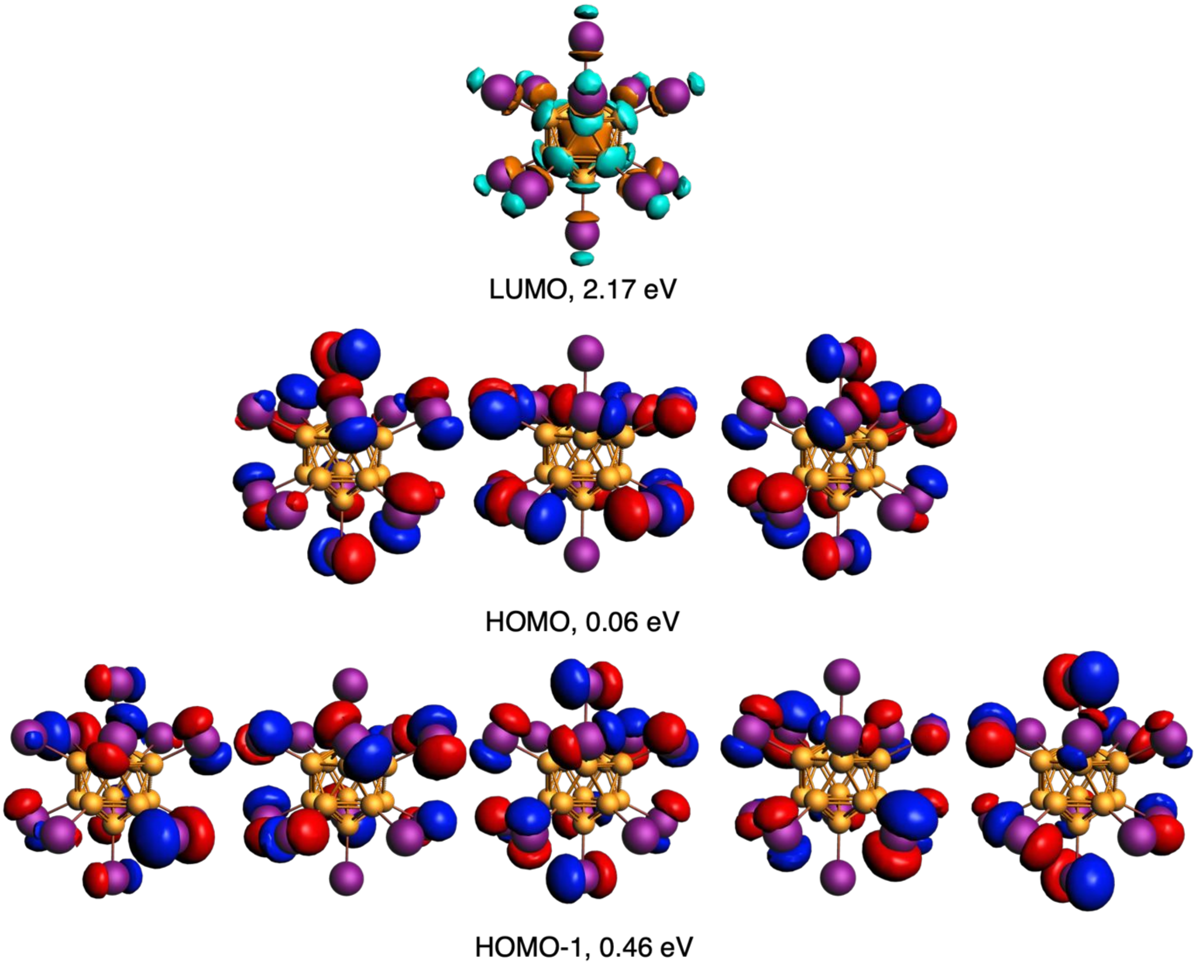

Figure S1. Frontier HOMO and LUMO molecular orbitals of $[B_{12}I_{12}]^{2-}$.

Table S1. GIMIC calculation parameters.

parameter	value
integration plane	xy (ivect=[1,0,0] and jvect=[0,1,0])
magnetic field orientation	z
cube: origin	[-16.0, -16.0, -16.0] bohr
cube: length	[32.0, 32.0, 32.0] bohr
grid points	[64, 64, 64]

a) b)

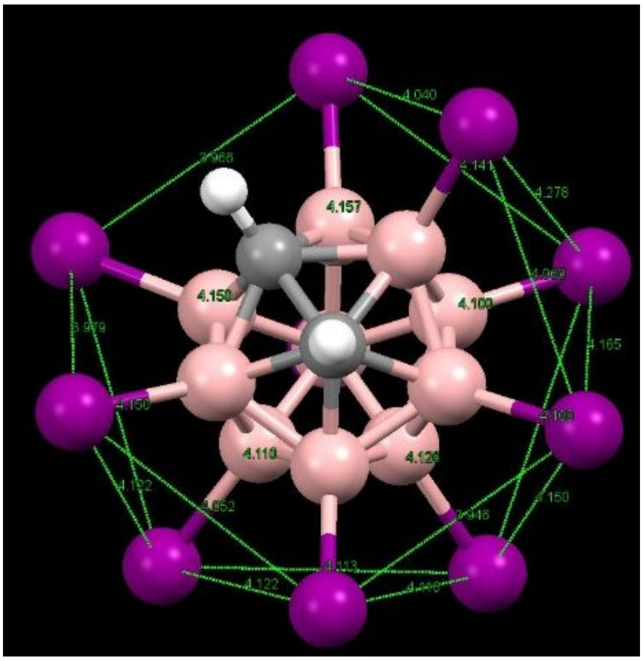

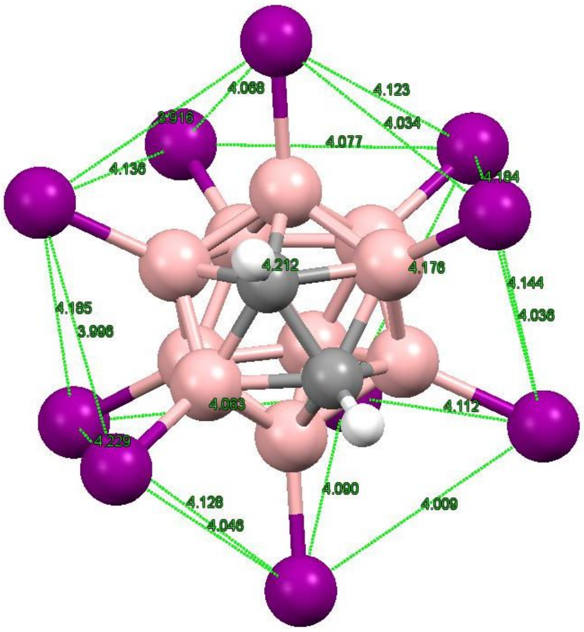

c)

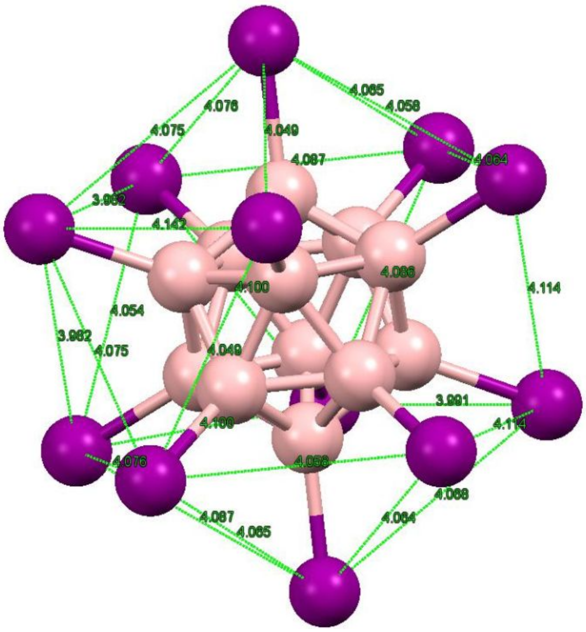

Figure S2. Crystal structures displaying the I–I distances. a) The neutral I_{10}-o-carborane (WUNDOZ) containing DMS and acetone (CEHWOC);[1] which are in the range 3.946-4.278 Å. b) the neutral I_{10}-*o*-carborane[2] in which the distances are in the range 3.916-4.212 Å and, c) $Na_2[B_{12}I_{12}]\cdot 8SO_2$ (the Na^+ cation and SO_2 molecules omitted for clarity)[3] in which the distances I–I are in the range 3.982 – 4.112 Å. The van der Waals radii of the I atom is 1.98 Å.[4]

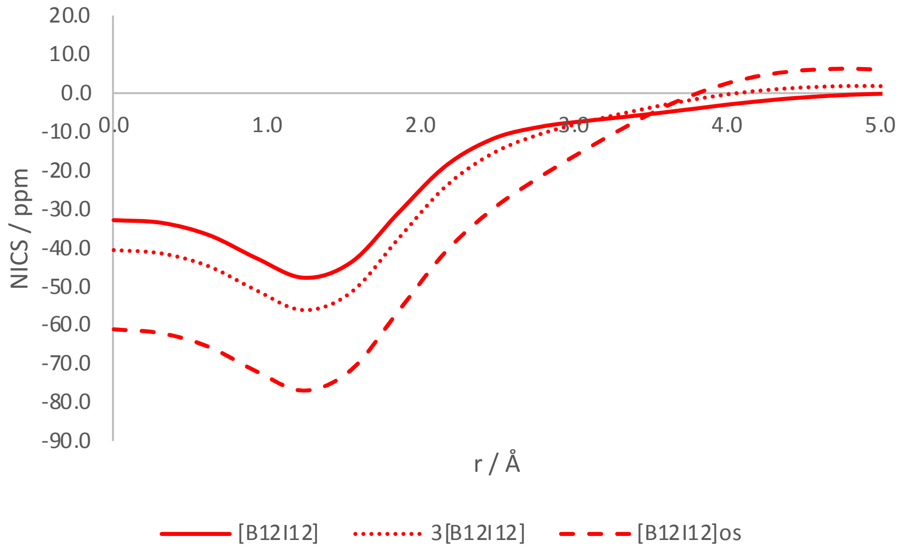

Figure S3. NICS scan (in ppm) from the center of the boron cluster to the middle of the closest I_3 three-membered ring for $[B_{12}I_{12}]$ cluster in singlet, open-shell singlet, and triplet states. Distances in Å.

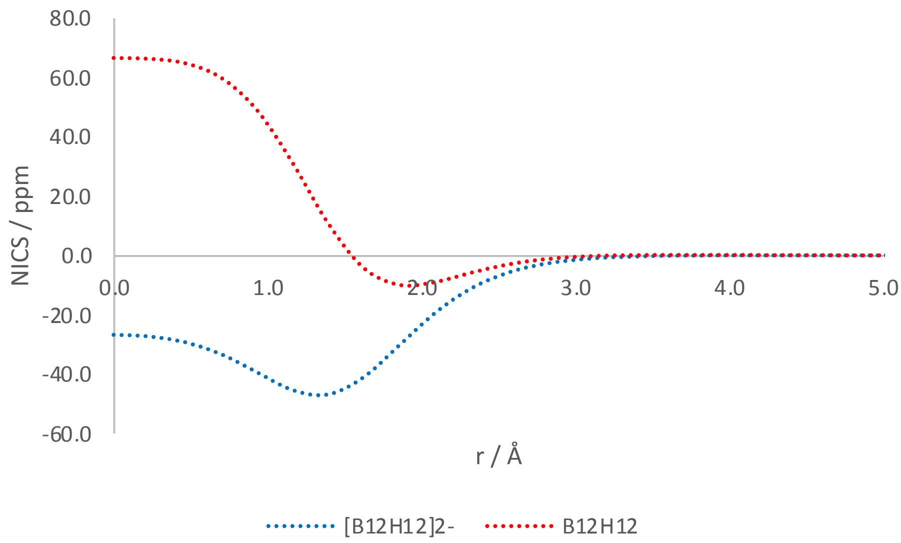

Figure S4. NICS scan (in ppm) from the center of the boron cluster to the middle of the closest H_3 three-membered ring for $[B_{12}H_{12}]^{2-}$ and $[B_{12}H_{12}]$ clusters. Distances in Å.

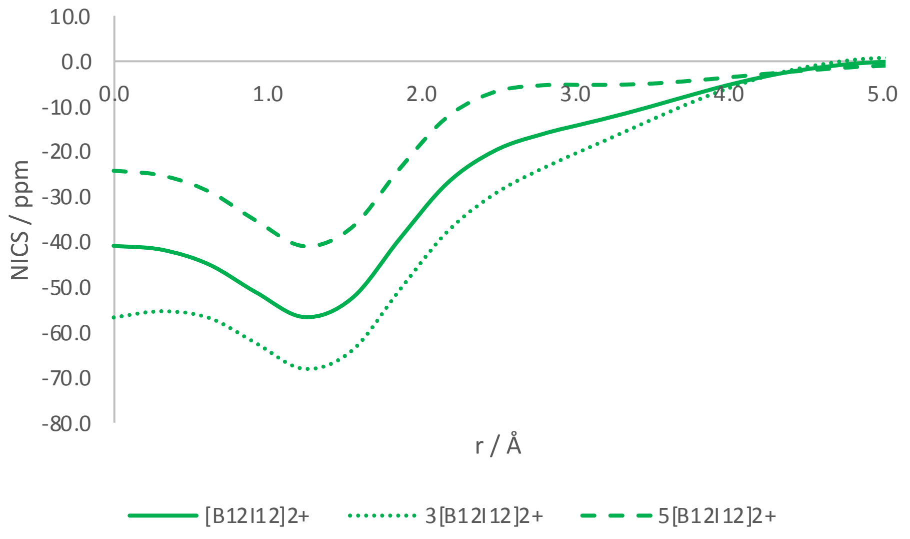

Figure S5. NICS scan (in ppm) from the center of the boron cluster to the middle of the closest I_3 three-membered ring for $[B_{12}I_{12}]^{2+}$ cluster in singlet, triplet, and quintet states. Distances in Å.

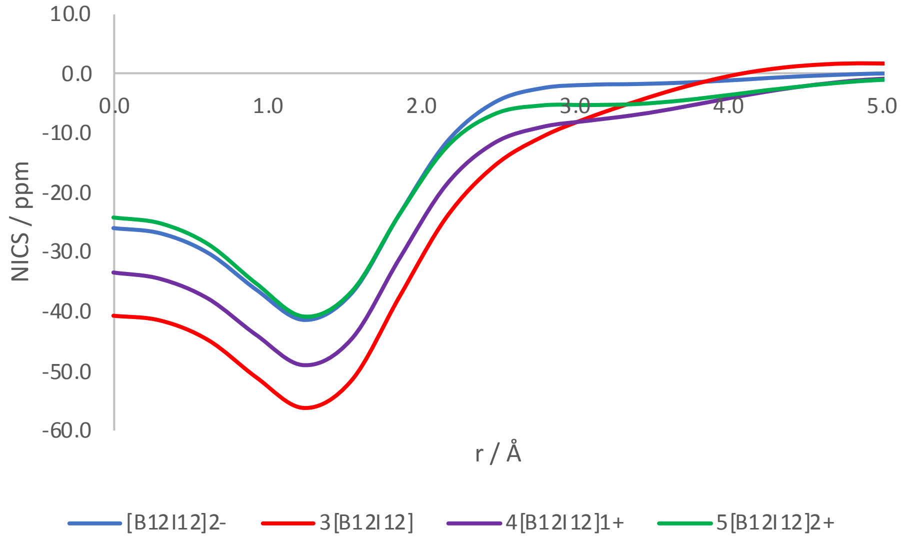

Figure S6. NICS scan (in ppm) from the center of the boron cluster to the middle of the closest I_3 three-membered ring for $[B_{12}I_{12}]^{2-}$ (singlet), $[B_{12}I_{12}]$ (triplet), $[B_{12}I_{12}]^{+}$ (quartet), and $[B_{12}I_{12}]^{2+}$ (quintet) clusters. Distances in Å.

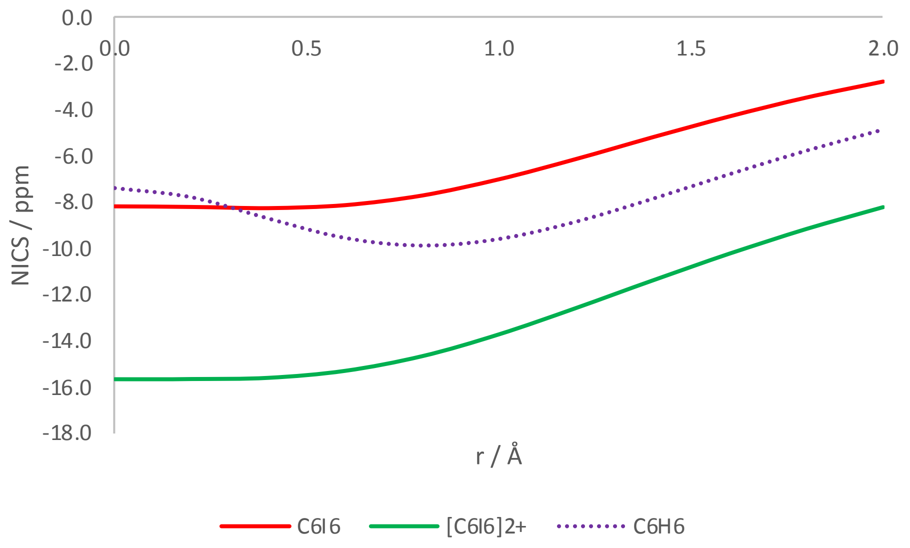

Figure S7. NICS scan (in ppm) perpendicular from the center of the ring for C_6I_6 and $[C_6I_6]^{2+}$ clusters. C_6H_6 has been added for comparison. Distances in Å.

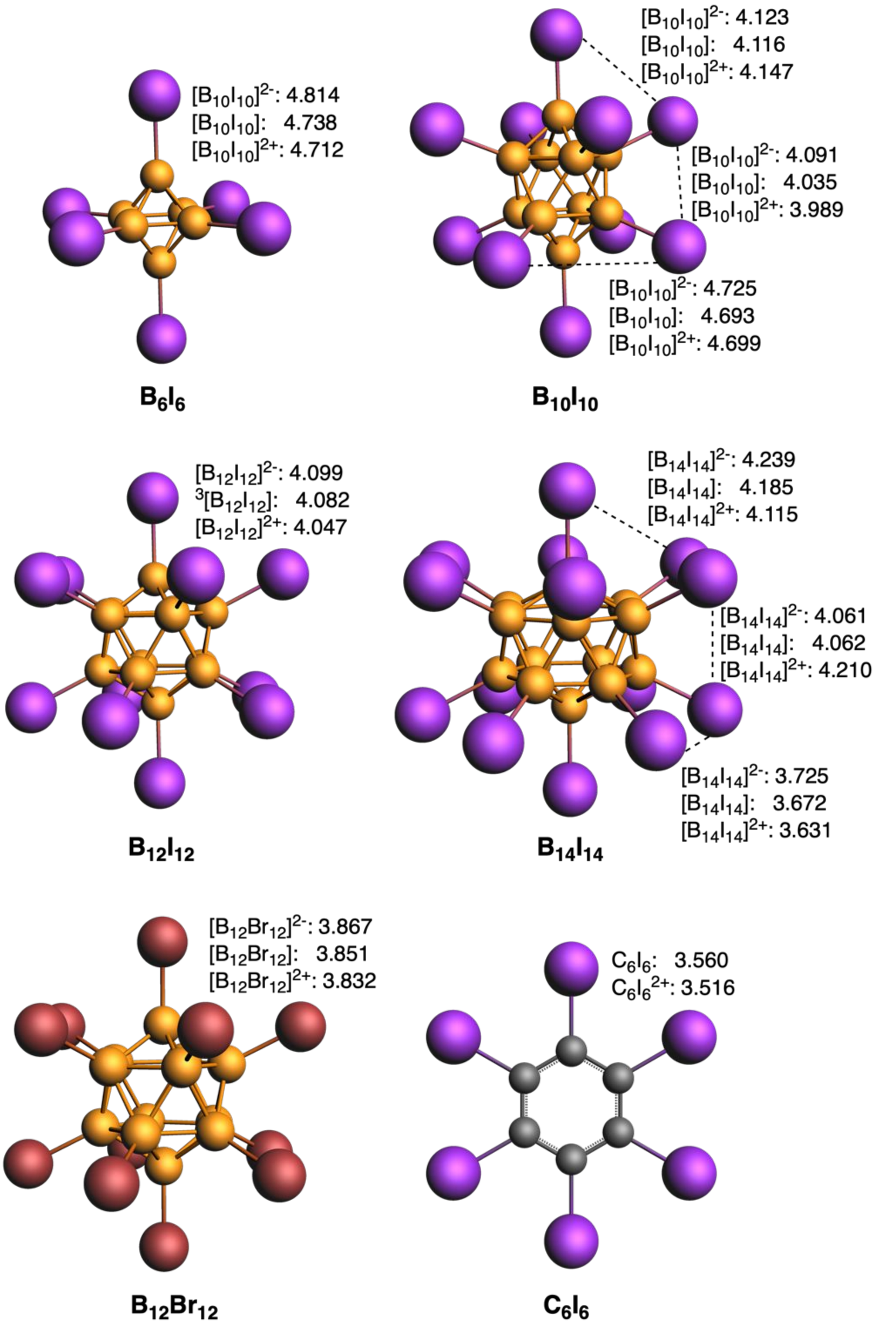

Figure S8. Shortest I···I bond length (in Å) of the boron clusters under analysis. $C_6I_6^{2+}$ has also been enclosed for comparison.

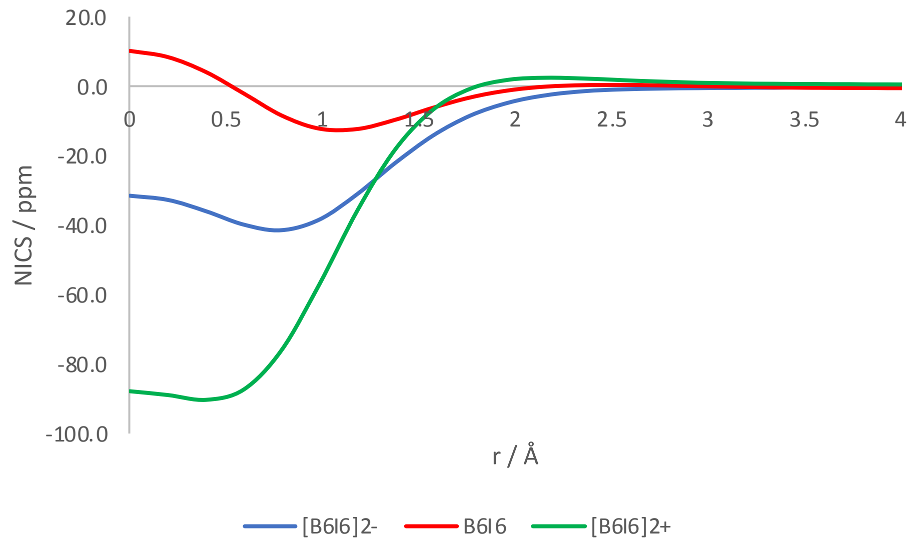

Figure S9. NICS scan (in ppm) from the center of the boron cluster to the middle of the closest I_3 three-membered ring for $[B_6I_6]^{2-}$, $[B_6I_6]$, and $[B_6I_6]^{2+}$ clusters. Distances in Å.

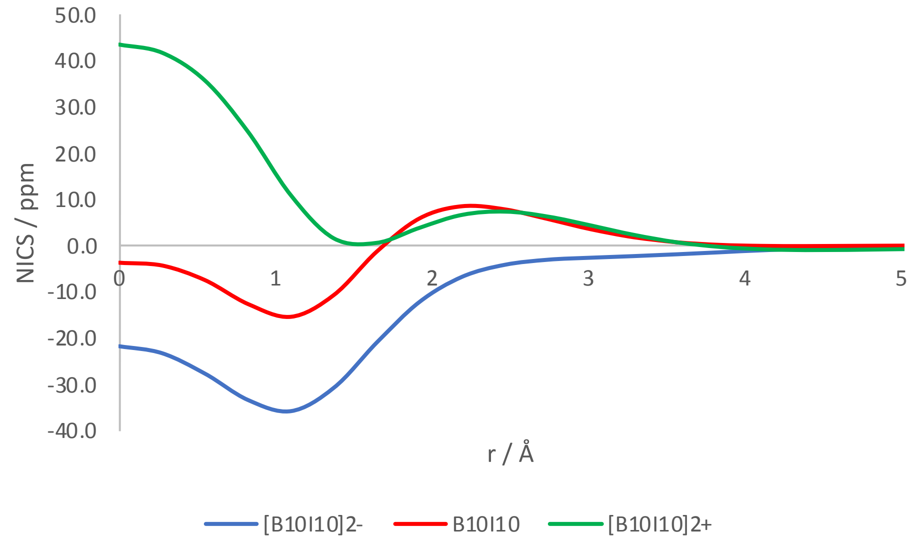

Figure S10. NICS scan (in ppm) from the center of the boron cluster to the middle of the closest I_3 three-membered ring for $[B_{10}I_{10}]^{2-}$, $[B_{10}I_{10}]$, and $[B_{10}I_{10}]^{2+}$ clusters. Distances in Å.

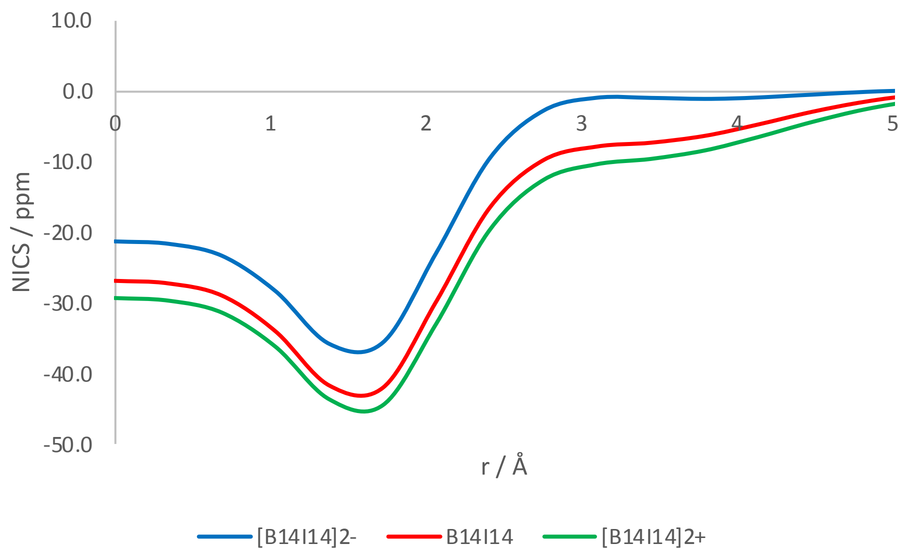

Figure S11. NICS scan (in ppm) from the center of the boron cluster to the middle of the closest I_3 three-membered ring for $[B_{14}I_{14}]^{2-}$, $[B_{14}I_{14}]$, and $[B_{14}I_{14}]^{2+}$ clusters. Distances in Å.

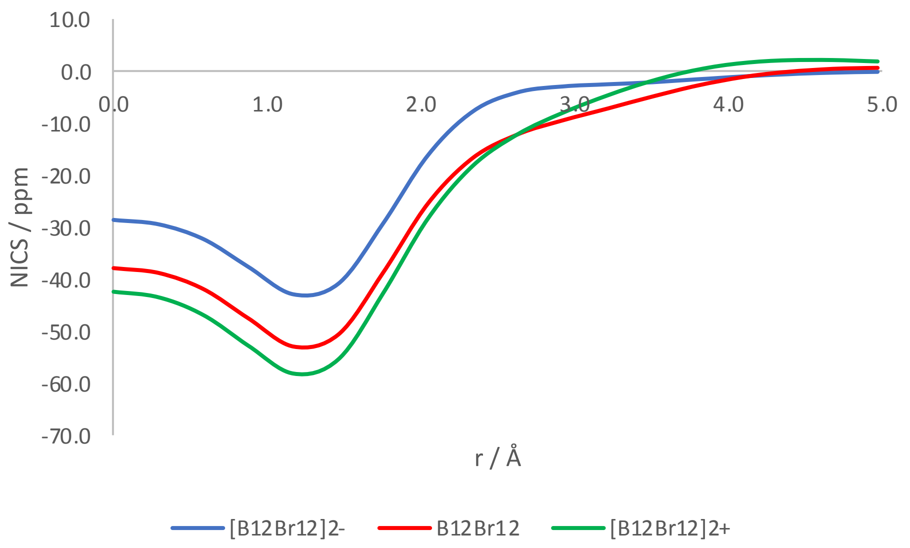

Figure S12. NICS scan (in ppm) from the center of the boron cluster to the middle of the closest I_3 three-membered ring for $[B_{12}Br_{12}]^{2-}$, $[B_{12}Br_{12}]$, and $[B_{12}Br_{12}]^{2+}$ clusters. Distances in Å.

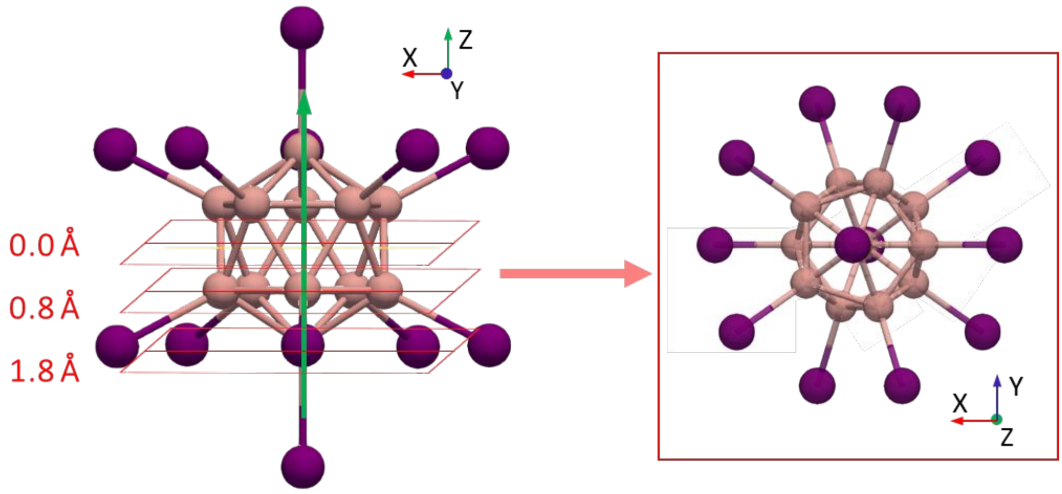

Figure S13. Orientation of the external magnetic field and perpendicular planes where the current density susceptibility has been sampled.

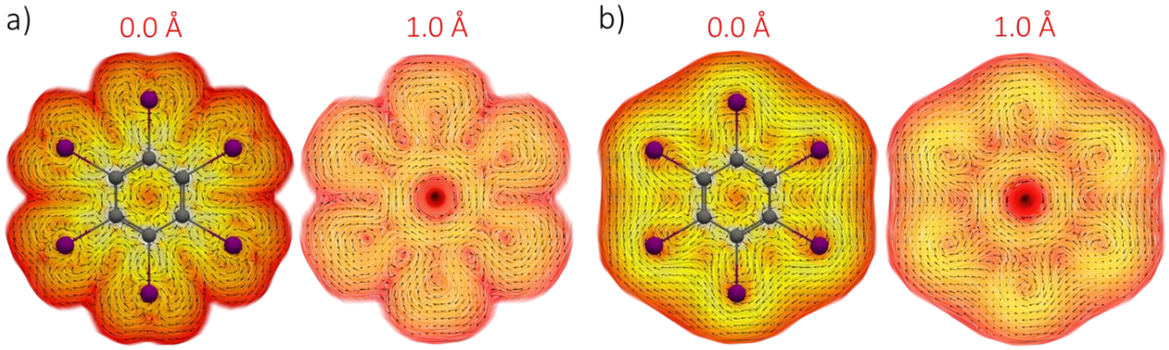

Figure S14. Current-density susceptibility of a) C_6I_6, and b) $C_6I_6^{2+}$ computed at the molecular plane, and the plane at 1 Å. The color scale corresponds to the strength of the modulus of the current-density susceptibility in the range of 0.0001 (red) to 0.4 (white) nA/T/Å^2.

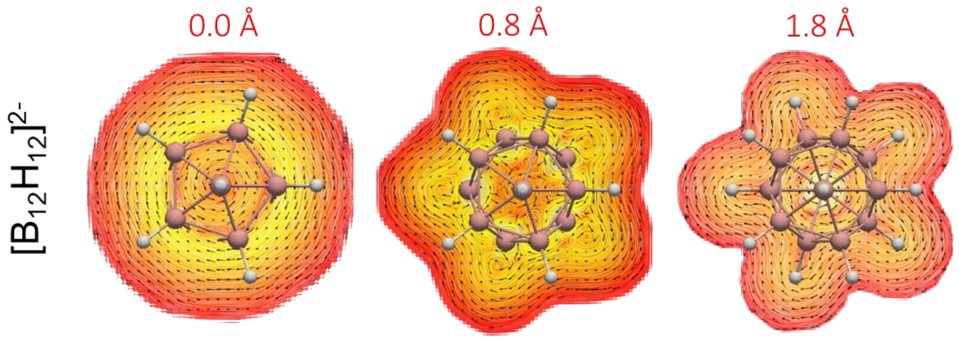

Figure S15. Current-density susceptibility of $[B_{12}H_{12}]^{2-}$ in the planes represented in Figure 1. The color scale corresponds to the strength of the modulus of the current-density susceptibility in the range of 0.0001 (red) to 0.4 (white) nA/T/Å^2.

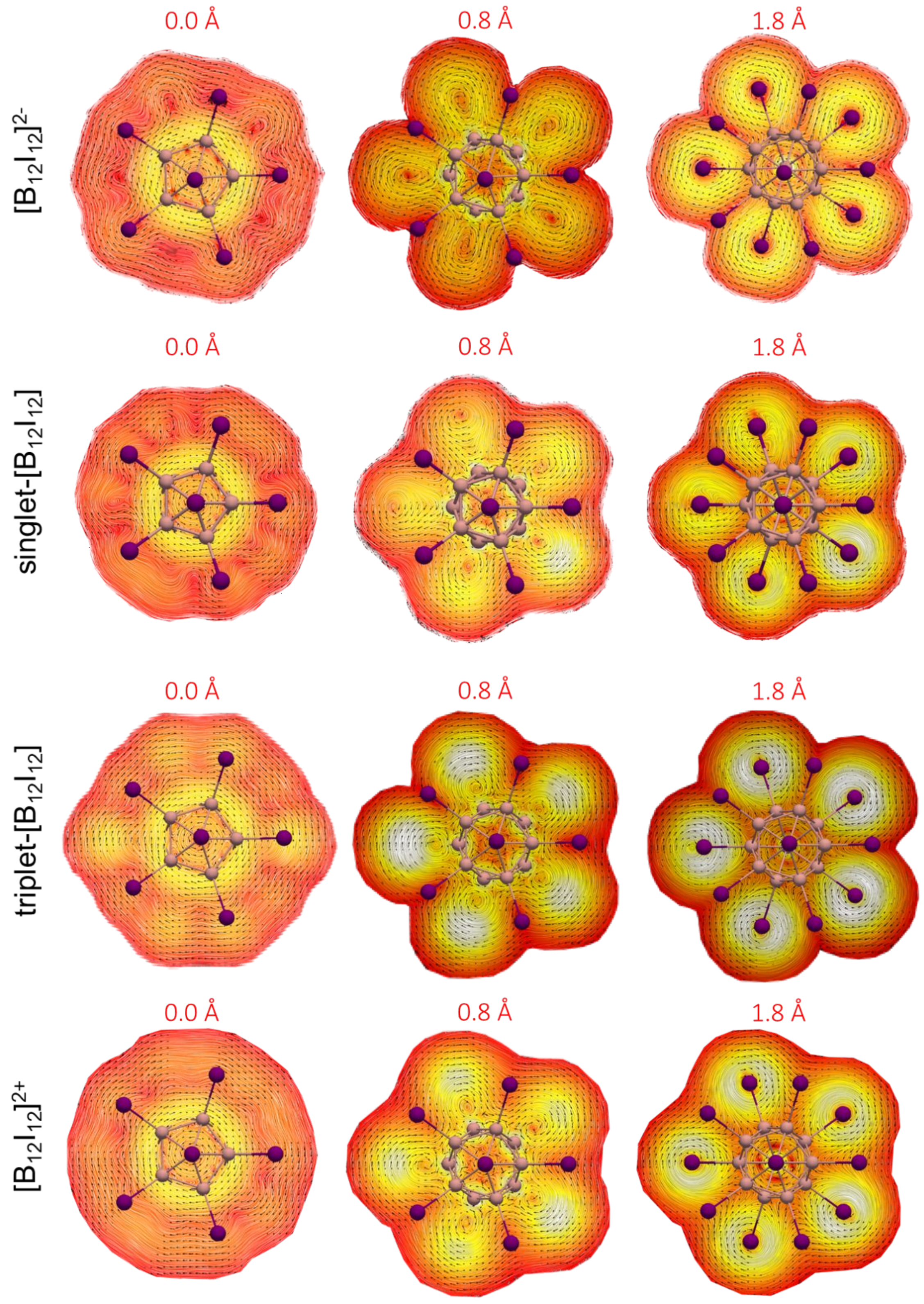

Figure S16. Current-density susceptibility of $[B_{12}I_{12}]^{2-}$, singlet and triplet $[B_{12}I_{12}]$, and $[B_{12}I_{12}]^{2+}$ in the planes represented in Figure 1. The color scale corresponds to the strength of the modulus of the current-density susceptibility in the range of 0.0001 (red) to 0.4 (white) nA/T/Å^2.

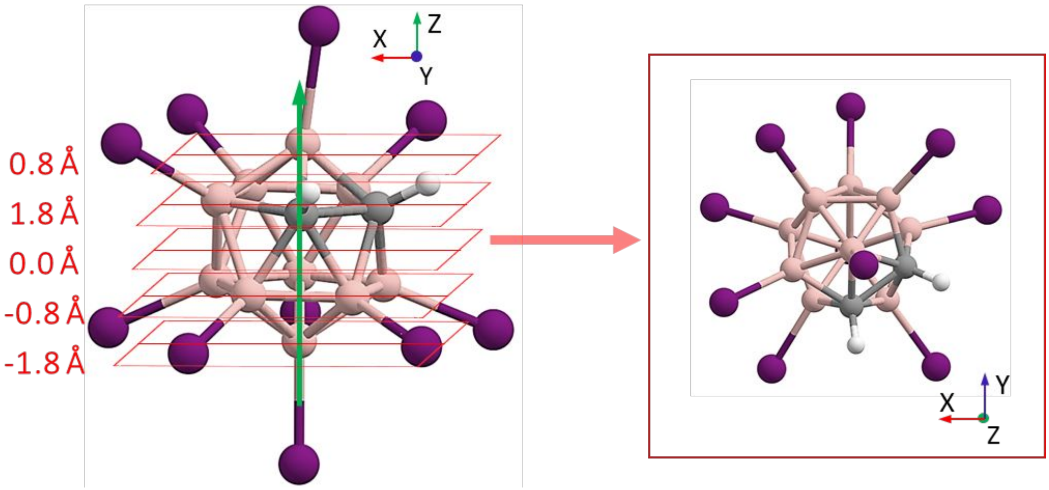

Figure S17. Orientation of the external magnetic field and perpendicular planes where the current density susceptibility has been sampled in the $^{X}[C_2B_{10}H_2I_{10}]^{N}$ species, where X = 1 or 3 and N = 0 or +2.

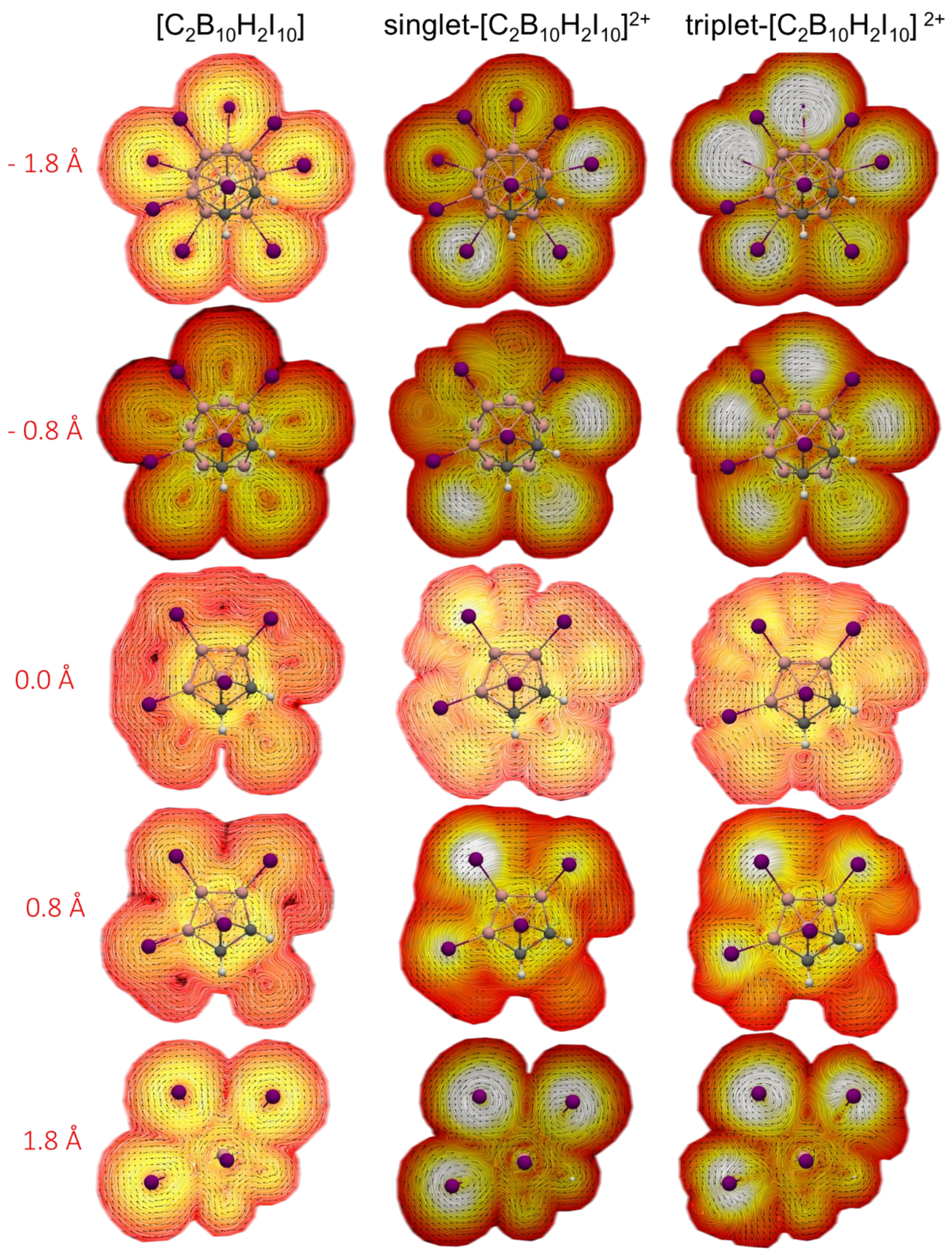

Figure S18. Current-density susceptibility of $[C_2B_{10}H_2I_{10}]$ and singlet and triplet $[C_2B_{10}H_2I_{10}]^{2+}$ in the planes represented in Figure S17. The color scale corresponds to the strength of the modulus of the current-density susceptibility in the range of 0.0001 (red) to 0.4 (white) nA/T/Å^2.

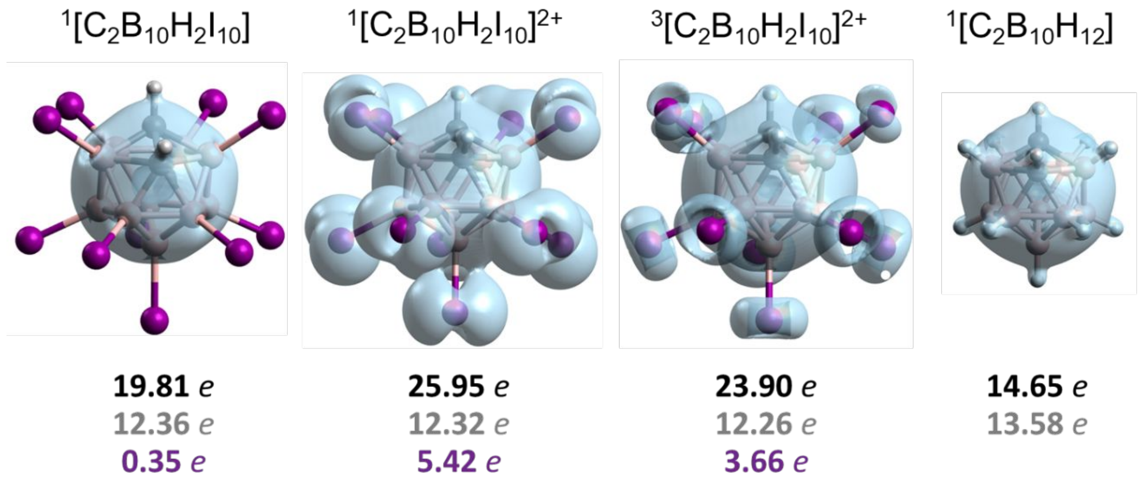

Figure S19. Isosurfaces (isocontour 0.007 e) of the electron density of delocalized bonds (EDDB). Numerical results correspond to $EDDB_G$ population of the whole system (black), boron and carbon (gray) and iodine (purple) atoms separately.

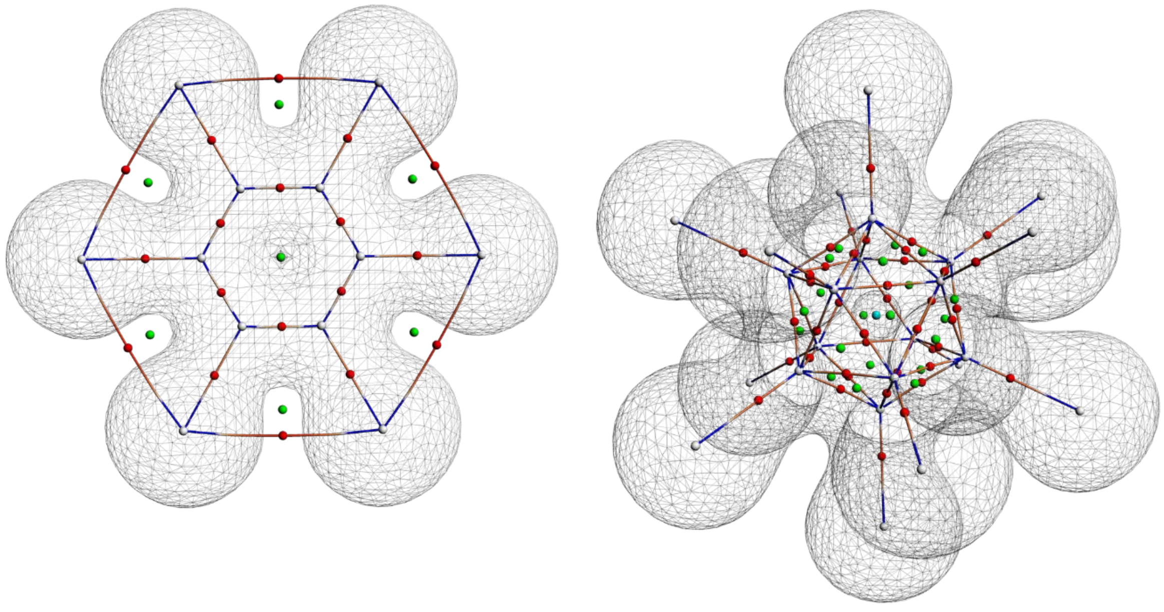

(a) (b)

Figure S20. QTAIM analysis of $C_6I_6^{+2}$ and $B_{12}I_{12}$.

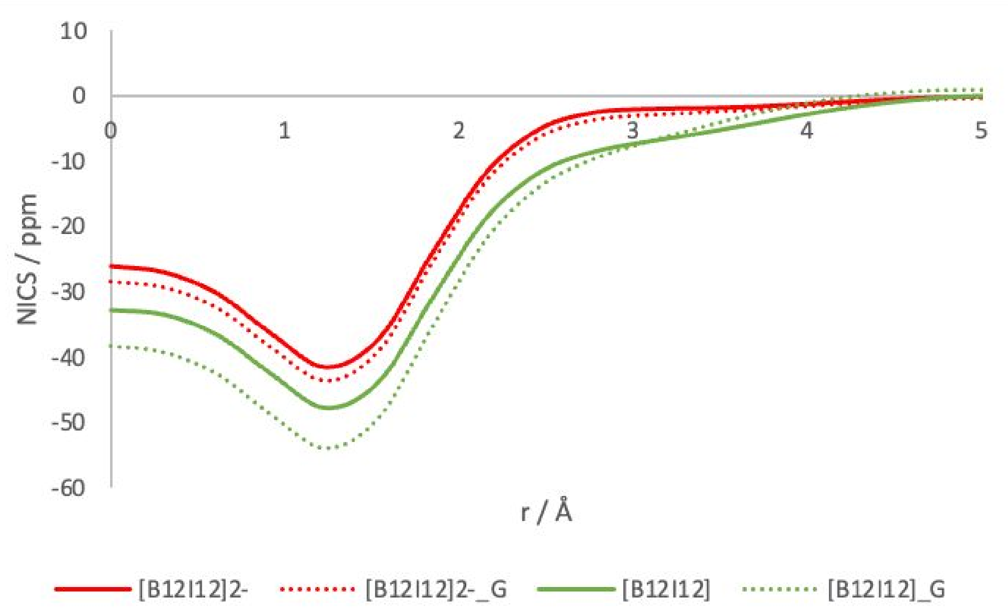

Figure S21. NICS scan (in ppm) from the center of the boron cluster to the middle of the closest I_3 three-membered ring for $[B_{12}I_{12}]^{2-}$ and singlet $[B_{12}I_{12}]$ clusters computed with the the ZORA-BLYP-D3(BJ)/TZ2P (solid line) and the B3LYP/6-311++G**~LANL2DZ (dotted line) methods. Distances in Å.

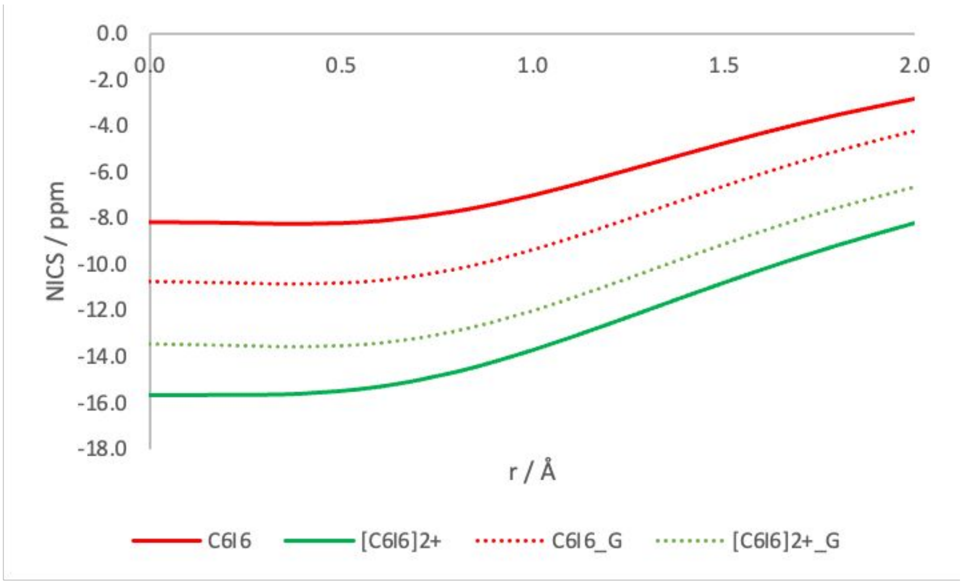

Figure S22. NICS scan (in ppm) from the center of the center of the benzene ring for B_6I_6 and singlet $[B_6I_6]^{2+}$ species computed with the the ZORA-BLYP-D3(BJ)/TZ2P (solid line) and the B3LYP/6-311++G**~LANL2DZ (dotted line) methods. Distances in Å.

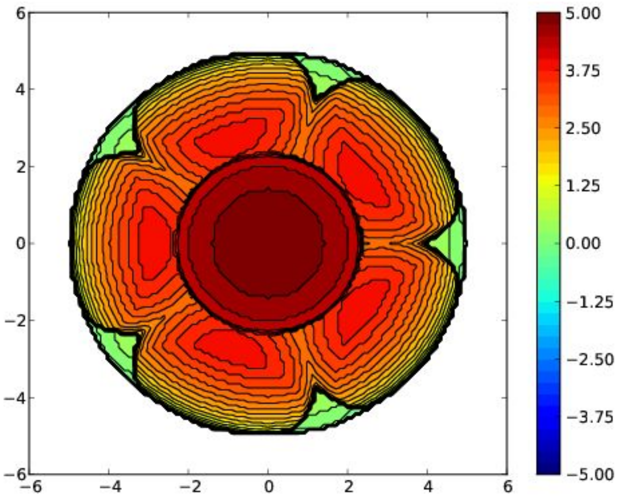

%V Free	%V Buried	% V Tot/V Ex
8.6	**91.4**	99.9

Quadrant	V f	V b	V t	%V f	%V b
SW	11.3	119.5	130.8	8.6	91.4
NW	11.3	119.5	130.8	8.6	91.4
NE	11.3	119.5	130.8	8.6	91.4
SE	11.3	119.5	130.8	8.6	91.4

Figure S23. $\%V_{Bur}$ and topographic steric maps in Å (XY plane) for $[B_{12}I_{12}]^{2-}$ using the center for each molecule or set of atoms under analysis, with a radius of 5.0 Å and with the Z axis defined by the 2 most opposed atoms of boron in $[B_{12}I_{12}]^{2-}$. The XY plane is perpendicular to the Z axis and contains the center of the molecule.

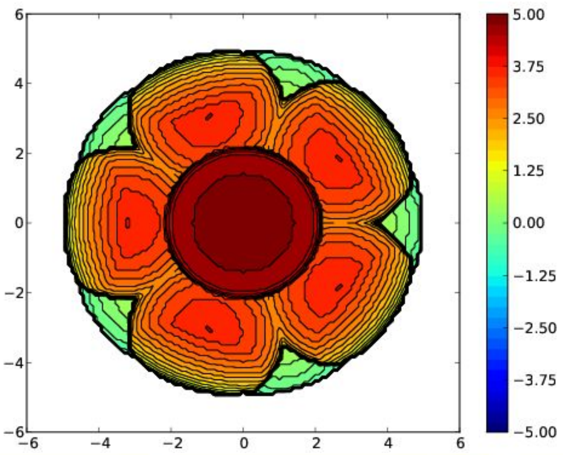

%V Free	%V Buried	% V Tot/V Ex
15.4	84.6	99.9

Quadrant	V f	V b	V t	%V f	%V b
SW	20.1	110.7	130.8	15.4	84.6
NW	20.1	110.7	130.8	15.4	84.6
NE	20.1	110.7	130.8	15.4	84.6
SE	20.1	110.7	130.8	15.4	84.6

Figure S24. $\%V_{Bur}$ and topographic steric maps in Å (XY plane) for $[B_{12}Br_{12}]^{2-}$ using the center for each molecule or set of atoms under analysis, with a radius of 5.0 Å and with the Z axis defined by the 2 most opposed atoms of boron in $[B_{12}Br_{12}]^{2-}$. The XY plane is perpendicular to the Z axis and contains the center of the molecule.

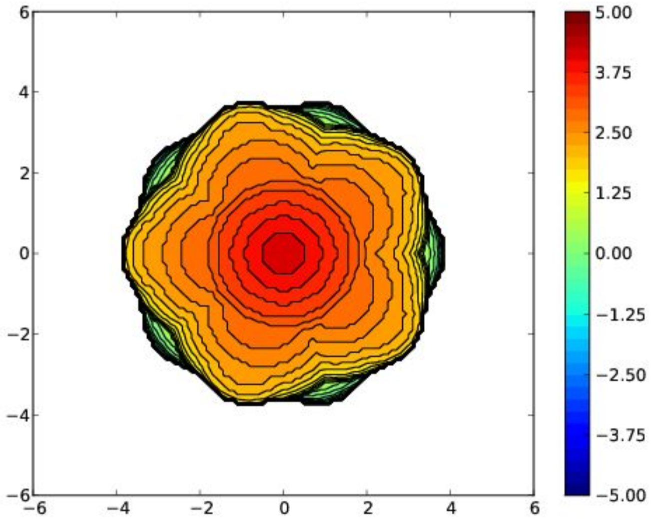

%V Free	%V Buried	% V Tot/V Ex
58.3	41.7	99.9

Quadrant	V f	V b	V t	%V f	%V b
SW	76.2	54.6	130.8	58.3	41.7
NW	76.2	54.6	130.8	58.3	41.7
NE	76.2	54.6	130.8	58.3	41.7
SE	76.2	54.6	130.8	58.3	41.7

Figure S25. $\%V_{Bur}$ and topographic steric maps in Å (XY plane) for $[B_{12}H_{12}]^{2-}$ using the center for each molecule or set of atoms under analysis, with a radius of 5.0 Å and with the Z axis defined by the 2 most opposed atoms of boron in $[B_{12}H_{12}]^{2-}$. The XY plane is perpendicular to the Z axis and contains the center of the molecule.

To ascertain the role of steric hindrance in different substituted *closo* icosahedral dodecaborates, iodine, is compared to bromine and hydrogen substituents. The 3 clusters, $[B_{12}I_{12}]^{2-}$, $[B_{12}Br_{12}]^{2-}$, and $[B_{12}H_{12}]^{2-}$ are studied with the SambVca2.1 package of Cavallo and coworkers, and the $\%V_{Bur}$ and steric maps are included in Figures S23-S25. Figure S26 reveals that the occupation of the plane with 5 halides is much higher in the case of iodine than bromine but only by a small difference. Thus, excluding the boron atoms, this plane suffers from the boron atoms of the same plane and the remaining 7, an occupation up to 72.5% for $[B_{12}I_{12}]^{2-}$, which is reduced only to 67.0% for $[B_{12}Br_{12}]^{2-}$, and to only 40.5 % by $[B_{12}H_{12}]^{2-}$.

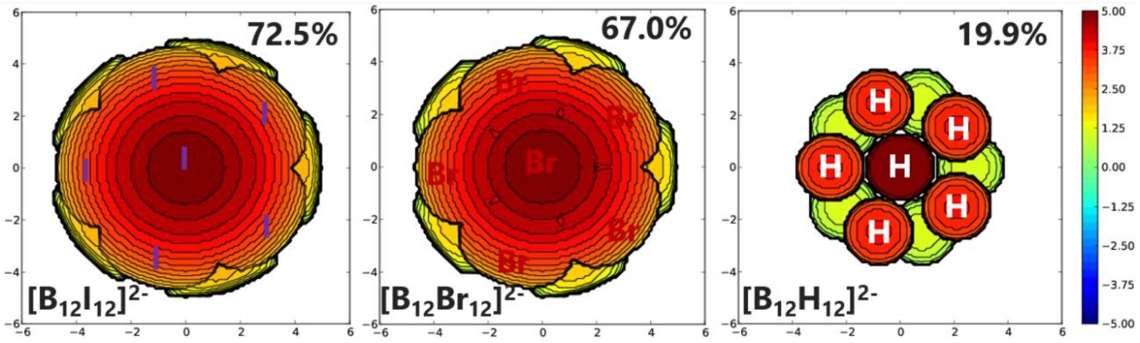

Figure S26. $\%V_{Bur}$ and topographic steric maps in Å (XY plane) using as centre the average of a set of 5 halides (or hydrogens) forming a plane as centre, with Z axis using the apical halide (or hydrogen) and any of the previous 5 halides (or hydrogens) to define the XZ axis for $[B_{12}I_{12}]^{2-}$, $[B_{12}Br_{12}]^{2-}$, and $[B_{12}H_{12}]^{2-}$.

Table S2. Electronic and Gibbs reaction energies (in kcal mol^{-1}) of selected homodesmotic reactions.

**Reaction**	Δ***E***	Δ***G***
$[C_6I_6]^{+2} + C_6H_6 \rightarrow [C_6I_5H]^{+2} + C_6H_5I$	17.5	15.6
$[C_6I_6]^{+2} + C_6H_6 \rightarrow [C_6I_4H_2]^{+2} + C_6H_4I_2$	37.5	35.8
$[C_6I_6]^{+2} + C_6H_6 \rightarrow [C_6H_3I_3]^{+2} + C_6H_3I_3$	37.1	34.1
$B_{12}I_{12} + [B_{12}H_{12}]^{2-} \rightarrow B_{12}I_{11}H + [B_{12}H_{11}I]^{2-}$	-22.1	-22.8
$B_{12}I_{12} + [B_{12}H_{12}]^{2-} \rightarrow B_{12}I_{10}H_2 + [B_{12}H_{10}I_2]^{2-}$	-32.6	-34.0
$B_{12}I_{12} + [B_{12}H_{12}]^{2-} \rightarrow B_{12}I_9H_3 + [B_9H_9I_3]^{2-}$	-38.4	-40.3
$B_{12}I_{12} + [B_{12}H_{12}]^{2-} \rightarrow B_{12}I_8H_4 + [B_9H_8I_4]^{2-}$	-41.5	-43.8
$B_{12}I_{12} + [B_{12}H_{12}]^{2-} \rightarrow B_{12}I_7H_5 + [B_9H_7I_5]^{2-}$	-40.6	-43.2
$B_{12}I_{12} + [B_{12}H_{12}]^{2-} \rightarrow B_{12}I_6H_6 + [B_9H_6I_6]^{2-}$	-37.5	-40.1
$^3[B_{12}I_{12}] + [B_{12}H_{12}]^{2-} \rightarrow {}^3[B_{12}I_6H_6] + [B_{12}H_6I_6]^{2-}$	-37.9	-40.7
$[B_{12}I_{12}]^{2+} + [B_{12}H_{12}]^{2-} \rightarrow [B_{12}I_6H_6]^{2+} + [B_{12}H_6I_6]^{2-}$	-2.8	-6.5

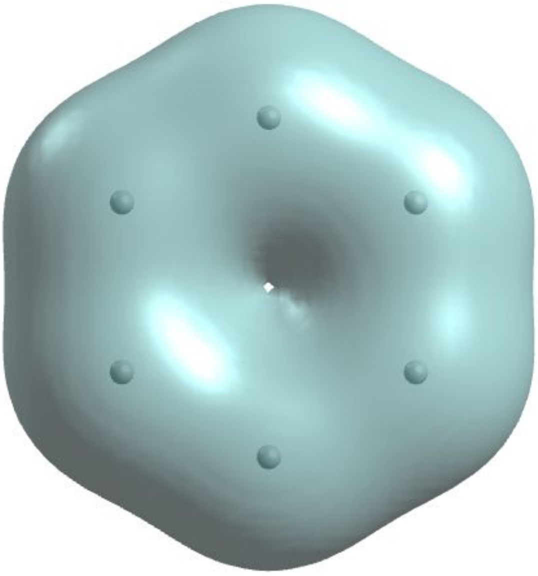

Figure S27. Isosurfaces (isocontour 0.007 e) of the electron density of delocalized bonds (EDDB) for H_6 model system.

Table S3. Cartesian coordinates and ADF total electronic energy (in parentheses and in kcal mol^{-1}) of $B_{12}I_{12}$ clusters under analysis computed at the ZORA-BLYP-D3(BJ)/TZ2P level of theory.

$[B_{12}I_{12}]^{2-}$ (-2371.2)

1	B	-0.89489939	1.23174959	0.76122464
2	I	-2.04968017	2.82167167	1.74542922
3	B	0.89489939	1.23174959	0.76122464
4	I	2.04968017	2.82167167	1.74542922
5	B	0.00000000	1.52251742	-0.76121046
6	I	0.00000000	3.48832011	-1.74380458
7	B	0.00000000	-0.00003233	-1.70225559
8	I	0.00000000	-0.00054551	-3.89990884
9	B	0.00000000	0.00003233	1.70225559
10	I	0.00000000	0.00054551	3.89990884
11	B	0.00000000	-1.52251742	0.76121046
12	I	0.00000000	-3.48832011	1.74380458
13	B	1.44800088	-0.47044072	0.76125265
14	I	3.31723357	-1.07653420	1.74542447
15	B	1.44800088	0.47044072	-0.76125265
16	I	3.31723357	1.07653420	-1.74542447
17	B	-1.44800088	-0.47044072	0.76125265
18	I	-3.31723357	-1.07653420	1.74542447
19	B	-1.44800088	0.47044072	-0.76125265
20	I	-3.31723357	1.07653420	-1.74542447
21	B	0.89489939	-1.23174959	-0.76122464
22	I	2.04968017	-2.82167167	-1.74542922
23	B	-0.89489939	-1.23174959	-0.76122464
24	I	-2.04968017	-2.82167167	-1.74542922

$B_{12}I_{12}$ (-2229.95)

1	B	1.19305500	0.82937500	0.89422200
2	I	2.72067400	1.89424600	2.04298100
3	B	1.19305500	0.82937500	-0.89422200
4	I	2.72067400	1.89424600	-2.04298100
5	B	-0.09071300	1.69219400	0.00000000
6	I	-0.18772500	3.87504100	0.00000000
7	B	-1.56729700	0.67342700	0.00000000
8	I	-3.57582200	1.54150300	0.00000000
9	B	1.56729700	-0.67342700	0.00000000
10	I	3.57582200	-1.54150300	0.00000000
11	B	0.09071300	-1.69219400	0.00000000
12	I	0.18772500	-3.87504100	0.00000000
13	B	0.51293300	-0.72946600	-1.44111100
14	I	1.19305500	-1.66298500	-3.29584200
15	B	-0.51293300	0.72946600	-1.44111100
16	I	-1.19305500	1.66298500	-3.29584200
17	B	0.51293300	-0.72946600	1.44111100
18	I	1.19305500	-1.66298500	3.29584200
19	B	-0.51293300	0.72946600	1.44111100

20	I	-1.19305500	1.66298500	3.29584200
21	B	-1.19305500	-0.82937500	-0.89422200
22	I	-2.72067400	-1.89424600	-2.04298100
23	B	-1.19305500	-0.82937500	0.89422200
24	I	-2.72067400	-1.89424600	2.04298100

$[B_{12}I_{12}]^{2+}$ (-1821.9)

1	B	1.43881500	0.10022000	0.88847900
2	I	3.29322000	0.22948700	2.03091500
3	B	1.43881500	0.10022000	-0.88847900
4	I	3.29322000	0.22948700	-2.03091500
5	B	0.79148700	1.50376800	0.00000000
6	I	1.82010900	3.42838800	0.00000000
7	B	-0.99258600	1.37929700	0.00000000
8	I	-2.27613400	3.14412400	0.00000000
9	B	0.99258600	-1.37929700	0.00000000
10	I	2.27613400	-3.14412400	0.00000000
11	B	-0.79148700	-1.50376800	0.00000000
12	I	-1.82010900	-3.42838800	0.00000000
13	B	0.06211300	-0.88831500	-1.43253100
14	I	0.14414700	-2.06394100	-3.26620200
15	B	-0.06211300	0.88831500	-1.43253100
16	I	-0.14414700	2.06394100	-3.26620200
17	B	0.06211300	-0.88831500	1.43253100
18	I	0.14414700	-2.06394100	3.26620200
19	B	-0.06211300	0.88831500	1.43253100
20	I	-0.14414700	2.06394100	3.26620200
21	B	-1.43881500	-0.10022000	-0.88847900
22	I	-3.29322000	-0.22948700	-2.03091500
23	B	-1.43881500	-0.10022000	0.88847900
24	I	-3.29322000	-0.22948700	2.03091500

$B_{12}I_{12}$ (triplet, -2235.7)

1	B	1.17992117	0.82337839	0.88878891
2	I	2.70145121	1.88738931	2.03596058
3	B	1.17992117	0.82337839	-0.88878891
4	I	2.70145121	1.88738931	-2.03596058
5	B	-0.09359575	1.68887285	0.00000000
6	I	-0.21179035	3.86832202	0.00000000
7	B	-1.55189983	0.67100262	0.00000000
8	I	-3.55573127	1.53510717	0.00000000
9	B	1.55189983	-0.67100262	0.00000000
10	I	3.55573127	-1.53510717	0.00000000
11	B	0.09359575	-1.68887285	0.00000000
12	I	0.21179035	-3.86832202	0.00000000
13	B	0.50821998	-0.72911768	-1.43734908
14	I	1.16348173	-1.67306471	-3.29243394
15	B	-0.50821998	0.72911768	-1.43734908
16	I	-1.16348173	1.67306471	-3.29243394
17	B	0.50821998	-0.72911768	1.43734908

18	I	1.16348173	-1.67306471	3.29243394
19	B	-0.50821998	0.72911768	1.43734908
20	I	-1.16348173	1.67306471	3.29243394
21	B	-1.17992117	-0.82337839	-0.88878891
22	I	-2.70145121	-1.88738931	-2.03596058
23	B	-1.17992117	-0.82337839	0.88878891
24	I	-2.70145121	-1.88738931	2.03596058

$[B_{12}I_{12}]^{2+}$ (triplet, -1826.8)

1	B	1.17483551	0.81892739	0.88563628
2	I	2.69579085	1.88019685	2.02853564
3	B	1.17483551	0.81892739	-0.88563628
4	I	2.69579085	1.88019685	-2.02853564
5	B	-0.09486047	1.68205385	0.00000000
6	I	-0.22067599	3.85768536	0.00000000
7	B	-1.54704049	0.66999367	0.00000000
8	I	-3.54706519	1.53720056	0.00000000
9	B	1.54704049	-0.66999367	0.00000000
10	I	3.54706519	-1.53720056	0.00000000
11	B	0.09486047	-1.68205385	0.00000000
12	I	0.22067599	-3.85768536	0.00000000
13	B	0.50723779	-0.72675106	-1.43268045
14	I	1.16203249	-1.67035597	-3.28447784
15	B	-0.50723779	0.72675106	-1.43268045
16	I	-1.16203249	1.67035597	-3.28447784
17	B	0.50723779	-0.72675106	1.43268045
18	I	1.16203249	-1.67035597	3.28447784
19	B	-0.50723779	0.72675106	1.43268045
20	I	-1.16203249	1.67035597	3.28447784
21	B	-1.17483551	-0.81892739	-0.88563628
22	I	-2.69579085	-1.88019685	-2.02853564
23	B	-1.17483551	-0.81892739	0.88563628
24	I	-2.69579085	-1.88019685	2.02853564

$[B_{12}I_{12}]^{2+}$ (quintet, -1822.7)

1	B	0.88737814	-1.22165814	0.75346777
2	I	2.01601117	-2.83934128	1.68670614
3	B	-0.88737814	-1.22165814	0.75346777
4	I	-2.01601117	-2.83934128	1.68670614
5	B	0.00000000	-1.50500764	-0.75066971
6	I	0.00000000	-3.45847632	-1.72757855
7	B	0.00000000	0.00098530	-1.68315407
8	I	0.00000000	-0.07500055	-3.86625456
9	B	0.00000000	-0.00098530	1.68315407
10	I	0.00000000	0.07500055	3.86625456
11	B	0.00000000	1.50500764	0.75066971
12	I	0.00000000	3.45847632	1.72757855
13	B	-1.43535874	0.46570083	0.75447565
14	I	-3.24133635	1.12257525	1.78948411
15	B	-1.43535874	-0.46570083	-0.75447565

16	I	-3.24133635	-1.12257525	-1.78948411
17	B	1.43535874	0.46570083	0.75447565
18	I	3.24133635	1.12257525	1.78948411
19	B	1.43535874	-0.46570083	-0.75447565
20	I	3.24133635	-1.12257525	-1.78948411
21	B	-0.88737814	1.22165814	-0.75346777
22	I	-2.01601117	2.83934128	-1.68670614
23	B	0.88737814	1.22165814	-0.75346777
24	I	2.01601117	2.83934128	-1.68670614

$[B_{12}I_{12}]^+$ (quartet, -2067.9)

1	B	1.41177844	-0.25739206	0.88687111
2	I	3.23446877	-0.58932845	2.03377089
3	B	1.41177844	-0.25739206	-0.88687111
4	I	3.23446877	-0.58932845	-2.03377089
5	B	1.12988905	1.25265328	0.00000000
6	I	2.58861618	2.87123689	0.00000000
7	B	-0.61522412	1.57087185	0.00000000
8	I	-1.40958809	3.59987817	0.00000000
9	B	0.61522412	-1.57087185	0.00000000
10	I	1.40958809	-3.59987817	0.00000000
11	B	-1.12988905	-1.25265328	0.00000000
12	I	-2.58861618	-2.87123689	0.00000000
13	B	-0.15910523	-0.87247346	-1.43488272
14	I	-0.36477170	-1.99883474	-3.28889708
15	B	0.15910523	0.87247346	-1.43488272
16	I	0.36477170	1.99883474	-3.28889708
17	B	-0.15910523	-0.87247346	1.43488272
18	I	-0.36477170	-1.99883474	3.28889708
19	B	0.15910523	0.87247346	1.43488272
20	I	0.36477170	1.99883474	3.28889708
21	B	-1.41177844	0.25739206	-0.88687111
22	I	-3.23446877	0.58932845	-2.03377089
23	B	-1.41177844	0.25739206	0.88687111
24	I	-3.23446877	0.58932845	2.03377089

Table S4. Cartesian coordinates and ADF total electronic energy (in parentheses and in kcal mol^{-1}) of supplementary compounds under analysis computed at the ZORA-BLYP-D3(BJ)/TZ2P level of theory.

C_6I_6 (-1277.7)

1	C	0.00000000	-1.41396500	0.00000000
2	C	1.22452900	-0.70698200	0.00000000
3	C	1.22452900	0.70698200	0.00000000
4	C	0.00000000	1.41396500	0.00000000
5	C	-1.22452900	0.70698200	0.00000000
6	C	-1.22452900	-0.70698200	0.00000000
7	I	3.08270100	-1.77979800	0.00000000
8	I	3.08270100	1.77979800	0.00000000
9	I	0.00000000	3.55959700	0.00000000
10	I	-3.08270100	1.77979800	0.00000000
11	I	-3.08270100	-1.77979800	0.00000000
12	I	0.00000000	-3.55959700	0.00000000

$[C_6I_6]^{2+}$ (-870.0)

1	C	0.00000000	-1.40074700	0.00000000
2	C	1.21308300	-0.70037400	0.00000000
3	C	1.21308300	0.70037400	0.00000000
4	C	0.00000000	1.40074700	0.00000000
5	C	-1.21308300	0.70037400	0.00000000
6	C	-1.21308300	-0.70037400	0.00000000
7	I	3.04527700	-1.75819200	0.00000000
8	I	3.04527700	1.75819200	0.00000000
9	I	0.00000000	3.51638300	0.00000000
10	I	-3.04527700	1.75819200	0.00000000
11	I	-3.04527700	-1.75819200	0.00000000
12	I	0.00000000	-3.51638300	0.00000000

$[C_6I_6]^{2-}$ (-1319.0)

1	C	0.00000000	-1.37832600	0.00000000
2	C	1.19366500	-0.68916300	0.00000000
3	C	1.19366500	0.68916300	0.00000000
4	C	0.00000000	1.37832600	0.00000000
5	C	-1.19366500	0.68916300	0.00000000
6	C	-1.19366500	-0.68916300	0.00000000
7	I	3.23662000	-1.86866400	0.00000000
8	I	3.23662000	1.86866400	0.00000000
9	I	0.00000000	3.73732700	0.00000000
10	I	-3.23662000	1.86866400	0.00000000
11	I	-3.23662000	-1.86866400	0.00000000
12	I	0.00000000	-3.73732700	0.00000000

C_6H_6 (-1680.7)

1	C	1.21186500	-0.69967100	0.00000000
2	C	1.21186500	0.69967100	0.00000000
3	C	0.00000000	1.39934100	0.00000000

4	C	-1.21186500	0.69967100	0.00000000
5	C	-1.21186500	-0.69967100	0.00000000
6	C	0.00000000	-1.39934100	0.00000000
7	H	2.15383700	1.24351900	0.00000000
8	H	0.00000000	2.48703700	0.00000000
9	H	-2.15383700	1.24351900	0.00000000
10	H	-2.15383700	-1.24351900	0.00000000
11	H	0.00000000	-2.48703700	0.00000000
12	H	2.15383700	-1.24351900	0.00000000

$[B_6I_6]^{2-}$ (-1137.7)

1	B	0.00000000	1.20867396	0.00000000
2	B	0.00000000	0.00000000	1.20867396
3	B	1.20867396	0.00000000	0.00000000
4	B	0.00000000	-1.20867396	0.00000000
5	B	0.00000000	0.00000000	-1.20867396
6	B	-1.20867396	0.00000000	0.00000000
7	I	0.00000000	3.40343015	0.00000000
8	I	0.00000000	-3.40343015	0.00000000
9	I	-3.40343015	0.00000000	0.00000000
10	I	0.00000000	0.00000000	3.40343015
11	I	3.40343015	0.00000000	0.00000000
12	I	0.00000000	0.00000000	-3.40343015

B_6I_6 (-1034.4)

1	B	0.00000000	1.22975806	0.00000000
2	B	0.00000000	0.00000000	1.22975806
3	B	1.22975806	0.00000000	0.00000000
4	B	0.00000000	-1.22975806	0.00000000
5	B	0.00000000	0.00000000	-1.22975806
6	B	-1.22975806	0.00000000	0.00000000
7	I	0.00000000	3.35032628	0.00000000
8	I	0.00000000	-3.35032628	0.00000000
9	I	-3.35032628	0.00000000	0.00000000
10	I	0.00000000	0.00000000	3.35032628
11	I	3.35032628	0.00000000	0.00000000
12	I	0.00000000	0.00000000	-3.35032628

$[B_6I_6]^{2+}$ (-599.3)

1	B	0.00000000	1.25762300	0.00000000
2	B	0.00000000	0.00000000	-1.28871700
3	B	-1.25762300	0.00000000	0.00000000
4	B	0.00000000	-1.25762300	0.00000000
5	B	0.00000000	0.00000000	1.28871700
6	B	1.25762300	0.00000000	0.00000000
7	I	0.00000000	3.33466800	0.00000000
8	I	0.00000000	-3.33466800	0.00000000
9	I	3.33466800	0.00000000	0.00000000
10	I	0.00000000	0.00000000	-3.32955500
11	I	-3.33466800	0.00000000	0.00000000

12	I	0.00000000	0.00000000	3.32955500

$[B_{10}I_{10}]^{2-}$ (-1943.3)

1	B	-1.20689206	-0.49995048	-0.76003591
2	B	0.49995048	-1.20689206	-0.76003591
3	B	-0.00000000	-0.00000000	-1.83383833
4	B	-0.49995048	1.20689206	-0.76003591
5	B	-1.20689206	0.49995048	0.76003591
6	B	0.49995048	1.20689206	0.76003591
7	B	0.00000000	0.00000000	1.83383833
8	B	1.20689206	-0.49995048	0.76003591
9	B	-0.49995048	-1.20689206	0.76003591
10	B	1.20689206	0.49995048	-0.76003591
11	I	-0.00000000	0.00000000	-4.01252049
12	I	3.08642609	1.27898546	-1.59679624
13	I	1.27898546	-3.08642609	-1.59679624
14	I	-3.08642609	-1.27898546	-1.59679624
15	I	-1.27898546	-3.08642609	1.59679624
16	I	3.08642609	-1.27898546	1.59679624
17	I	-0.00000000	0.00000000	4.01252049
18	I	1.27898546	3.08642609	1.59679624
19	I	-3.08642609	1.27898546	1.59679624
20	I	-1.27898546	3.08642609	-1.59679624

$B_{10}I_{10}$ (-1804.2)

1	B	-1.21696064	-0.50413854	-0.74840141
2	B	0.50413854	-1.21696064	-0.74840141
3	B	0.00000000	0.00000000	-1.86783128
4	B	-0.50413854	1.21696064	-0.74840141
5	B	-1.21696064	0.50413854	0.74840141
6	B	0.50413854	1.21696064	0.74840141
7	B	-0.00000000	-0.00000000	1.86783128
8	B	1.21696064	-0.50413854	0.74840141
9	B	-0.50413854	-1.21696064	0.74840141
10	B	1.21696064	0.50413854	-0.74840141
11	I	0.00000000	0.00000000	-4.00441187
12	I	3.06533992	1.27045821	-1.56837188
13	I	1.27045821	-3.06533992	-1.56837188
14	I	-3.06533992	-1.27045821	-1.56837188
15	I	-1.27045821	-3.06533992	1.56837188
16	I	3.06533992	-1.27045821	1.56837188
17	I	-0.00000000	-0.00000000	4.00441187
18	I	1.27045821	3.06533992	1.56837188
19	I	-3.06533992	1.27045821	1.56837188
20	I	-1.27045821	3.06533992	-1.56837188

$[B_{10}I_{10}]^{2+}$ (-1382.3)

1	B	-1.23154814	-0.51051057	-0.73915911
2	B	0.51051057	-1.23154814	-0.73915911
3	B	-0.00000000	-0.00000000	-1.91458509

4	B	-0.51051057	1.23154814	-0.73915911
5	B	-1.23154814	0.51051057	0.73915911
6	B	0.51051057	1.23154814	0.73915911
7	B	0.00000000	0.00000000	1.91458509
8	B	1.23154814	-0.51051057	0.73915911
9	B	-0.51051057	-1.23154814	0.73915911
10	B	1.23154814	0.51051057	-0.73915911
11	I	-0.00000000	0.00000000	-4.01679514
12	I	3.06924958	1.27299058	-1.53524076
13	I	1.27299058	-3.06924958	-1.53524076
14	I	-3.06924958	-1.27299058	-1.53524076
15	I	-1.27299058	-3.06924958	1.53524076
16	I	3.06924958	-1.27299058	1.53524076
17	I	-0.00000000	0.00000000	4.01679514
18	I	1.27299058	3.06924958	1.53524076
19	I	-3.06924958	1.27299058	1.53524076
20	I	-1.27299058	3.06924958	-1.53524076

$[B_{14}I_{14}]^{2-}$ (-2693.6)

1	B	-1.72503455	-0.33152880	0.77064092
2	B	-1.14951875	1.32724452	0.77134596
3	B	0.57494953	1.65864795	0.77194536
4	B	1.72492032	0.33153687	0.77084472
5	B	1.14940891	-1.32721857	0.77150218
6	B	-0.57505318	-1.65864589	0.77188741
7	I	-2.44007679	2.81549558	1.78678017
8	I	1.21763848	3.51865895	1.79078320
9	I	3.65981524	0.70157178	1.78552997
10	I	2.43987548	-2.81549497	1.78706798
11	I	-1.21790620	-3.51860850	1.79068577
12	I	-3.66000537	-0.70158075	1.78510859
13	B	-1.65861201	0.57499562	-0.77201035
14	B	-0.33149076	1.72496356	-0.77077556
15	B	1.32728047	1.14946693	-0.77134752
16	B	1.65870241	-0.57500472	-0.77181226
17	B	0.33157493	-1.72496542	-0.77072427
18	B	-1.32719319	-1.14945390	-0.77149863
19	I	-0.70143081	3.65991309	-1.78537284
20	I	2.81562205	2.43991896	-1.78677516
21	I	3.51872553	-1.21775471	-1.79053372
22	I	0.70178944	-3.65988932	-1.78526334
23	I	-2.81552871	-2.43984995	-1.78707232
24	I	-3.51860315	1.21762134	-1.79094060
25	B	-0.00005683	-0.00001748	-1.61228070
26	B	0.00007949	0.00000913	1.61228035
27	I	0.00004074	-0.00002335	-3.80867110
28	I	-0.00002936	0.00001860	3.80867061

$B_{14}I_{14}$ (-2580.5)

1	B	-1.70405004	-0.32765412	0.76741672

2	B	-1.13553751	1.31109466	0.76812768
3	B	0.56798647	1.63871258	0.76848114
4	B	1.70393246	0.32765979	0.76763126
5	B	1.13542728	-1.31106663	0.76828917
6	B	-0.56808563	-1.63871294	0.76841771
7	I	-2.40448840	2.77586967	1.79474631
8	I	1.20136370	3.46905166	1.79717798
9	I	3.60695766	0.69314782	1.79402831
10	I	2.40429496	-2.77588460	1.79499673
11	I	-1.20158500	-3.46900847	1.79709773
12	I	-3.60711679	-0.69313207	1.79368417
13	B	-1.63868008	0.56803215	-0.76854705
14	B	-0.32761830	1.70397226	-0.76755681
15	B	1.31112620	1.13548798	-0.76812740
16	B	1.63876863	-0.56804345	-0.76834108
17	B	0.32769241	-1.70397829	-0.76750600
18	B	-1.31104217	-1.13547638	-0.76828739
19	I	-0.69302227	3.60703041	-1.79390978
20	I	2.77595377	2.40437811	-1.79474748
21	I	3.46910690	-1.20147946	-1.79696172
22	I	0.69325468	-3.60702613	-1.79380955
23	I	-2.77580600	-2.40440351	-1.79499511
24	I	-3.46896786	1.20146821	-1.79731009
25	B	-0.00005095	-0.00001956	-1.62135269
26	B	0.00006973	0.00000838	1.62135243
27	I	0.00015631	-0.00001983	-3.80208680
28	I	-0.00015678	0.00001829	3.80208642

$[B_{14}I_{14}]^{2+}$ (-2166.3)

1	B	1.72232500	-0.22393300	-0.77353300
2	B	1.05492700	1.37912200	-0.77448600
3	B	-0.66718300	1.60284200	-0.77527200
4	B	-1.72225200	0.22394300	-0.77369000
5	B	-1.05485500	-1.37911100	-0.77460400
6	B	0.66725600	-1.60283000	-0.77523300
7	I	2.20603700	2.88312300	-1.88435000
8	I	-1.39521700	3.35172800	-1.88374900
9	I	-3.60083200	0.46796800	-1.88299000
10	I	-2.20586000	-2.88309500	-1.88459800
11	I	1.39539400	-3.35170100	-1.88366800
12	I	3.60100900	-0.46794200	-1.88266000
13	B	1.60279900	0.66721400	0.77533200
14	B	0.22390100	1.72228200	0.77363400
15	B	-1.37915200	1.05488500	0.77448900
16	B	-1.60287300	-0.66722600	0.77517300
17	B	-0.22397500	-1.72229400	0.77358800
18	B	1.37908000	-1.05489700	0.77460100
19	I	0.46786500	3.60090700	1.88287200
20	I	-2.88319600	2.20593500	1.88435500
21	I	-3.35180300	-1.39532000	1.88354100

22	I	-0.46804500	-3.60093400	1.88277800
23	I	2.88302200	-2.20596000	1.88459200
24	I	3.35162600	1.39529000	1.88387600
25	B	-0.00007900	-0.00001200	1.64956300
26	B	0.00007800	0.00001200	-1.64956300
27	I	-0.00017600	-0.00002800	3.82120500
28	I	0.00017600	0.00002800	-3.82120500

$[B_{12}Br_{12}]^{2-}$ (-2544.8)

1	B	1.26169945	0.70605982	0.89357842
2	Br	2.72999628	1.52772072	1.93439117
3	B	1.26169945	0.70605982	-0.89357842
4	Br	2.72999628	1.52772072	-1.93439117
5	B	0.07379596	1.69808585	0.00000000
6	Br	0.16034416	3.67458852	0.00000000
7	B	-1.48583196	0.82535242	0.00000000
8	Br	-3.21516446	1.78641042	0.00000000
9	B	1.48583196	-0.82535242	0.00000000
10	Br	3.21516446	-1.78641042	0.00000000
11	B	-0.07379596	-1.69808585	0.00000000
12	Br	-0.16034416	-3.67458852	0.00000000
13	B	0.43641941	-0.77973921	-1.44571875
14	Br	0.94499398	-1.68774814	-3.12811187
15	B	-0.43641941	0.77973921	-1.44571875
16	Br	-0.94499398	1.68774814	-3.12811187
17	B	0.43641941	-0.77973921	1.44571875
18	Br	0.94499398	-1.68774814	3.12811187
19	B	-0.43641941	0.77973921	1.44571875
20	Br	-0.94499398	1.68774814	3.12811187
21	B	-1.26169945	-0.70605982	-0.89357842
22	Br	-2.72999628	-1.52772072	-1.93439117
23	B	-1.26169945	-0.70605982	0.89357842
24	Br	-2.72999628	-1.52772072	1.93439117

$B_{12}Br_{12}$ (-2364.1)

1	B	1.16936800	0.85262100	0.89108100
2	Br	2.53491900	1.84926900	1.93274600
3	B	1.16936800	0.85262100	-0.89108100
4	Br	2.53491900	1.84926900	-1.93274600
5	B	-0.12812800	1.68390400	0.00000000
6	Br	-0.25527300	3.66754500	0.00000000
7	B	-1.57593700	0.63623100	0.00000000
8	Br	-3.41660700	1.38143100	0.00000000
9	B	1.57593700	-0.63623100	0.00000000
10	Br	3.41660700	-1.38143100	0.00000000
11	B	0.12812800	-1.68390400	0.00000000
12	Br	0.25527300	-3.66754500	0.00000000
13	B	0.52729700	-0.71522300	-1.43592500
14	Br	1.16936800	-1.54877400	-3.12242000
15	B	-0.52729700	0.71522300	-1.43592500

16	Br	-1.16936800	1.54877400	-3.12242000
17	B	0.52729700	-0.71522300	1.43592500
18	Br	1.16936800	-1.54877400	3.12242000
19	B	-0.52729700	0.71522300	1.43592500
20	Br	-1.16936800	1.54877400	3.12242000
21	B	-1.16936800	-0.85262100	-0.89108100
22	Br	-2.53491900	-1.84926900	-1.93274600
23	B	-1.16936800	-0.85262100	0.89108100
24	Br	-2.53491900	-1.84926900	1.93274600

$[B_{12}Br_{12}]^{2+}$ (-1866.2)

1	B	0.00568900	1.43909100	0.89059700
2	Br	0.01217800	3.11952100	1.94717200
3	B	0.00568900	1.43909100	-0.89059700
4	Br	0.01217800	3.11952100	-1.94717200
5	B	-1.42619300	0.89445300	0.00000000
6	Br	-3.08746500	1.98664000	0.00000000
7	B	-1.43323900	-0.88314700	0.00000000
8	Br	-3.10388800	-1.96106400	0.00000000
9	B	1.43323900	0.88314700	0.00000000
10	Br	3.10388800	1.96106400	0.00000000
11	B	1.42619300	-0.89445300	0.00000000
12	Br	3.08746500	-1.98664000	0.00000000
13	B	0.88625100	-0.00340500	-1.43680100
14	Br	1.92077500	-0.00762200	-3.13311700
15	B	-0.88625100	0.00340500	-1.43680100
16	Br	-1.92077500	0.00762200	-3.13311700
17	B	0.88625100	-0.00340500	1.43680100
18	Br	1.92077500	-0.00762200	3.13311700
19	B	-0.88625100	0.00340500	1.43680100
20	Br	-1.92077500	0.00762200	3.13311700
21	B	-0.00568900	-1.43909100	-0.89059700
22	Br	-0.01217800	-3.11952100	-1.94717200
23	B	-0.00568900	-1.43909100	0.89059700
24	Br	-0.01217800	-3.11952100	1.94717200

References

[1] F. Teixidor, G. Barberà, C. Viñas, R. Sillanpää, R. Kivekäs, *Inorg. Chem.* **2006**, *45*, 3496-3498.
[2] A. V. Puga, F. Teixidor, R. Sillanpää, R. Kivekäs, C. Viñas, *Chem. Eur. J.* **2009**, *15*, 9764-9772.
[3] S. E. Hollow, T. C. Johnstone, *Chem. Commun.* **2022**, *58*, 2375-2378.
[4] A. Bondi, *J. Phys. Chem.* **1964**, *68*, 441-451.
